# Real-time community detection in full social networks on a laptop

**DOI:** 10.1371/journal.pone.0188702

**Published:** 2018-01-17

**Authors:** Benjamin Paul Chamberlain, Josh Levy-Kramer, Clive Humby, Marc Peter Deisenroth

**Affiliations:** 1 Department of Computing, Imperial College London, London, United Kingdom; 2 Starcount Insights, London, United Kingdom; Tongji University, CHINA

## Abstract

For a broad range of research and practical applications it is important to understand the allegiances, communities and structure of key players in society. One promising direction towards extracting this information is to exploit the rich relational data in digital social networks (the social graph). As global social networks (e.g., Facebook and Twitter) are very large, most approaches make use of distributed computing systems for this purpose. Distributing graph processing requires solving many difficult engineering problems, which has lead some researchers to look at single-machine solutions that are faster and easier to maintain. In this article, we present an approach for analyzing full social networks on a standard laptop, allowing for interactive exploration of the communities in the locality of a set of user specified query vertices. The key idea is that the aggregate actions of large numbers of users can be compressed into a data structure that encapsulates the edge weights between vertices in a derived graph. Local communities can be constructed by selecting vertices that are connected to the query vertices with high edge weights in the derived graph. This compression is robust to noise and allows for interactive queries of local communities in real-time, which we define to be less than the average human reaction time of 0.25s. We achieve single-machine real-time performance by compressing the neighborhood of each vertex using minhash signatures and facilitate rapid queries through Locality Sensitive Hashing. These techniques reduce query times from hours using industrial desktop machines operating on the full graph to milliseconds on standard laptops. Our method allows exploration of strongly associated regions (i.e., communities) of large graphs in real-time on a laptop. It has been deployed in software that is actively used by social network analysts and offers another channel for media owners to monetize their data, helping them to continue to provide free services that are valued by billions of people globally.

## Introduction

Social media data provides a record of global human interactions at a scale that is hitherto unprecedented. These interactions are an invaluable resource for analyzing social allegiances, discovering entities with shared interests, and identifying key players in communities. The size of social networks (e.g., Facebook or Twitter), with hundreds of millions of users and billions of connections, makes social media analysis hard, and often requires big computing and data centers to extract the desired information.

In this article, we focus on the problem of discovering communities in full social networks, such as Facebook and Twitter in real-time. We define real-time to be less than the average human reaction time, which is 0.25s [1]. On social media, people connect with other people (e.g., by being a “friend” or “following” somebody) forming a *social graph*. Discovering communities in the social graph has a large number of practical applications, which include

- Security, where analysts explore a network looking for groups of potential adversaries;
- Social sciences, where queries can establish the important relationships between individuals of interest;
- E-commerce, where queries reveal related products or users;
- Marketing, where companies seek to optimize advertising channels or celebrity endorsement portfolios.

These applications do not disrupt user experience in the way that sponsored links or feed advertising do, offering an alternative means for social media providers to continue to offer free services.

**Fig 1:**
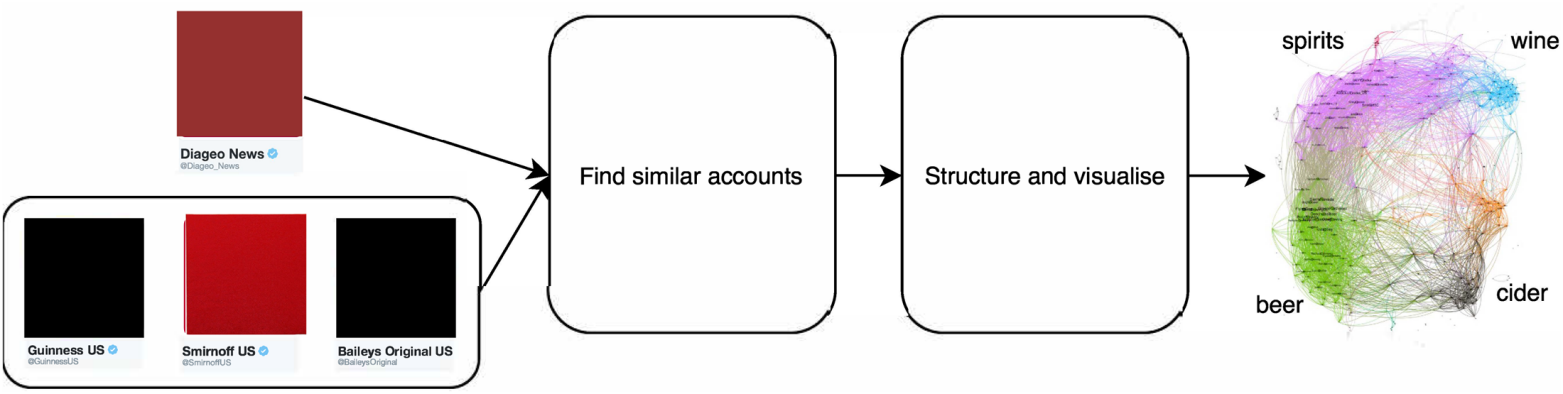
Example application of a real-time community detection system. Diageo want to explore the market (competitors, customers, associations etc.) around their brand. They feed in information about themselves (“seeds”). In this example the seeds are the company itself (Diageo) and some of their major brands (Smirnoff, Baileys and Guinness). Our systems finds accounts that are similar/related to the seeds and then structures the similar accounts into communities.

A concrete example of a commercial application of community detection using Twitter data is strategic support to a company that wants to trade in a new geographic region. To enter a new geography a company must understand the competitors, customers and marketing channels in that region. Our system allows them to extract this valuable information by providing the Twitter handles for their existing products, key people, brands and endorsers. In real-time, they can receive the accounts closely related to their company filtered by that market. The output is automatically structured into groups (communities) such as media titles, sportspeople and other related companies. Analysts examine the results, and our real-time system allows them to interactively explore the network by changing the input accounts. We show a high-level illustration for an alcoholic drinks brand in Fig 1.

In this article, we consider community detection with three practical restrictions: **(1) computations need to be in real-time, (2) can run on a single laptop and (3) can handle very large graphs.** A system that satisfies these constraints is vital to analysts who wish to interactively explore and analyze community structures in large graphs. To achieve our objective of a large-scale, real-time community detection system that runs on a laptop, we combine global and local approaches to community detection: First, we use local community detection to identify an interesting region of a graph, and then we apply global community detection to help understand that region. In the experimental section we describe several concrete applications, one of which is understanding the local community around and the structure of the US Republican party. In this case an analyst could query the Twitter graph with the accounts ‘Donald Trump’, ‘Marco Rubio’, ‘Ted Cruz’, ‘Ben Carson’ and ‘Jeb Bush’ (data from December 2015). The system finds the local community around these query vertices, which in this case happens to be the right wing of US politics. The returned results indicate the sub-communities that exist within the US right wing. On seeing these, an analyst may decide that they are specifically interested in one subgroup. In this case one such subgroup are gun / rifle lobbyists and could run a follow up query using ‘the national rifles association’, ‘gun owners of America’ and ‘the national association of gun rights’, which are three accounts surfaced by the original query.

## Background, key challenges and contribution

A social media network is a structure that comprises a graph and a collection of metadata describing the vertices *V* and/or edges *E* of the graph. A *community* has no formal mathematical definition, but is generally agreed to be is a collection of vertices *C* ⊂ *V* that share many more edges than would be expected from a random subset of vertices [2]. In the context of the Twitter graph, a vertex *V* is a Twitter account and an (undirected) edge *E* between *V*_*i*_, *V*_*j*_ exists if *V*_*i*_ Follows *V*_*j*_ or *V*_*j*_ Follows *V*_*i*_. Here, and in the remainder of the paper, we capitalize Follows when describing the Twitter specific meaning of this word. A community might be the set of Twitter accounts belonging to machine learning researchers. In addition to the Twitter graph, the Twitter *network* also includes metadata associated with the accounts (e.g., name, description) and edges (e.g., creation time, direction).

In this article, we develop a robust real-time algorithm for community detection for which we focus exclusively on the properties of the graph, i.e., no metadata of the Twitter/Facebook network is required. In particular, we propose that robust associations between social network accounts can be reached by considering the similarity of their neighborhood graphs [3]. The *neighborhood graph* of a vertex consists of the set of all vertices that are directly connected to it, irrespective of the edge direction. Neighborhood graphs of Digital Social Networks (DSNs) can be very large; In Twitter, the largest have almost 100 million members (as of June 2016).

Our proposition of exploiting the neighborhood graphs relies on the existence of *homophily* in social networks: The homophily principle states that people with similar attributes are more likely to form relationships [4]. An important assumption that we make is that the DSNs we study are representative of the underlying social networks. In reality the underlying networks and the DSNs themselves are multiplex (Twitter users retweet and comment in addition to Following). Interactions occur over multiple channels and modeling only a simplex network is known to introduce inaccuracies [5]. We choose this approach as communication density in the additional layers of our experimental networks are orders of magnitude more sparse than the layer we study (the median number of retweets from a Twitter user in our dataset is zero, comments are even more sparse). Accordingly, we feel that the assumption that social media accounts with similar neighborhood graphs are likely to have similar attributes is valid for these datasets.

Our real-time system exhibits the following properties:

1. It produces high quality communities from very noisy data. Quality is defined using the community axioms described in the community axioms section [6].
2. It is robust to failure and does not require engineering support.
3. It is parsimonious with the time of its users. Specifically we mean returning results in real-time and having sub-linear time complexity with the size of the network (Discussed in detail in the Time Complexity section).

The first constraint leads us to use the neighborhood graph as the unit of comparison between vertices. The neighborhood graph is generated by the actions of large numbers of independent users in contrast to features like text content or group memberships, which are usually controlled by a single user. The second requirement leads us to search for a solution that does not require large computing resources and can run on a laptop. The third property prescribes a real-time system.

Currently, no tool exists that provides real-time analysis of large graphs on a single commodity machine. Existing methods to analyze local community structure in large graphs either rely on distributed computing facilities or incur excessive run-times making them impractical for exploratory and interactive work [7, 8].

In this article, we introduce a novel real-time analysis tool for detecting communities in large graphs using only a laptop. We focus on a dataset of 700 million Twitter users. However, our approach is more generally applicable as it relies only on the general graph structure, not on Twitter-specific data. We provide additional results using Facebook data to demonstrate the general applicability.

For real-time community detection, we need to solve two core challenges:

1. The graph must be fit into the memory of a laptop.
2. Many neighborhood graphs containing up to 100 million vertices must be compared in milliseconds.

To address the first challenge, we compress the neighborhood graphs. We achieve this by using fixed-length minhash signatures, which vastly reduces the size of the graph. Choosing appropriate length minhash signatures squeezes the graph into memory. Minhash signatures allow for an efficient comparison of neighborhood graphs by means of the Jaccard similarity, which can be efficiently computed for minhash signatures. To solve the second challenge and achieve real-time querying we use the elements of the minhash signatures as the basis to build a Locality Sensitive Hashing (LSH) data structure. LSH facilitates querying of similar accounts in constant time.

The combination of minhashing and LSH allows analysts to enter an account or a set of accounts and in milliseconds receive the set of most related accounts. From this set we use the minhash signatures to rapidly construct a weighted graph and apply the WALKTRAP community detection algorithm before visualizing the results [9].

Our novel system is practical and combines proven techniques in an innovative way: (1) Minhashing and LSH are applied to the neighborhood graph [10, 11]; minhashing is normally only used to compare very similar sets. (2) We show that minhashing is effective for community detection when applied to a broad range of neighborhood graph similarities. (3) We develop an agglomerative clustering algorithm and an original update procedure for minhash signatures in this setting. The novel combination of these techniques allows our system to perform robust real-time community detection on a laptop using graphs that exceed 100 million vertices.

The contributions of this article are:

1. We establish that robust associations between social media users can be determined by means of the Jaccard similarity of their neighborhood graphs.
2. We show that the approximations implicit in minhashing and LSH minimally degrade performance and allow querying of very large graphs in real-time.
3. System design and evaluation: We have designed and evaluated an end-to-end system for extracting data from social media providers, compressing the data into a form where it can be efficiently queried in real-time.
4. We demonstrate how queries can be applied to a range of problems in graph analysis, e.g., understanding the structure of industries, allegiances within political parties and the public image of a brand.

Our code base is publicly available at https://github.com/melifluos/LSH-community-detection, which includes instructions for replicating our experiments.

### Related work

Existing approaches to large-scale, efficient, community detection have three flavors: More efficient community detection algorithms, innovative ways to perform processing on large graphs and data structures for graph compression and search. Table 1 shows related approaches to this problem and which of our three constraints they satisfy. The table highlights that there is currently only one way to do real-time community detection on large graphs (Twitter WTF [19]). However, this method requires a large computing infrastructure and does not run on a laptop. Algorithms that do run on a laptop are not real-time capable and may not even perform community detection on large graphs.

**Table 1:** Comparison of related work. SCM stands for runs on a Single Commodity Machine.

Method	Real-time	Large graphs	SCM
Modularity optimization [12]	✘	✘	✔
WALKTRAP [9]	✘	✘	✔
INFOMAP [13]	✘	✘	✔
Louvain method [14]	✘	✔	✔
BigClam [6]	✘	✔	✔
Graphlab [15]	✘	✔	✘
Pregel [16]	✘	✔	✘
Surfer [17]	✘	✔	✘
Graphci [18]	✘	✔	✔
Twitter WTF [19]	✔	✔	✘
LEMON [20]	✘	✔	✔
Our Method	✔	✔	✔

#### Community detection algorithms

Community detection methods have been developed in areas as diverse as neuronal firing [21], electron spin alignment [22] and social models [6]. [23] and [24] both provide excellent and detailed overviews of the diverse community detection literature. Approaches can be broadly categorized into local and global methods.

Global methods assign every vertex to a community, usually by partitioning the vertices. Many highly innovative schemes have been developed to do this. Modularity optimization [12] is one of the best known. Modularity is a metric used to evaluate the quality of a graph partition. Communities are determined by selecting the partition that maximizes the modularity. An alternative to modularity was developed in [9] who innovatively applied random walks on the graph to define communities as regions in which walkers become trapped (WALKTRAP). In [13], random walks are combined with efficient coding theory to produce INFOMAP, a technique that provides a new perspective on community detection: Communities are defined as the structural sub-units that facilitate the most efficient encoding of information flow through a network. All three methods are well optimized for their motivating networks, but do not scale to modern DSNs.

The availability of data from the Web, DSNs and services like Wikipedia has focused research attention on algorithms that scale. An early success was the Louvain method that allowed modularity optimization to be used to perform community detection on large graphs (they report 100 million vertices and 1 billion edges). However, the method was not intended to be real-time, and the reported 152 minute runtime on a biopteron 2.2k with 24GB of memory is too slow to achieve real-time performance, even allowing for nearly a decade of hardware advances [14]. Another noteworthy technique applied to very large graphs is Bigclam [6]. Bigclam is a multiple membership model, meaning that each vertex can be assigned to more than one community. This differs from the Louvain method, which assigns each vertex to a single community. As vertices can belong to more than one community, Bigclam can be said to detect overlapping communities. However, in common with the Louvain method, Bigclam is not a real-time algorithm that could facilitate interactive exploration of social networks.

In contrast to global community detection methods, local methods do not assign every vertex to a community. Instead they find vertices that are in the same community as a set of input vertices (seeds). For this reason they are normally faster than global methods. Local community detection methods were originally developed as crawling strategies to cope with the rapidly expanding web-graph [25]. Following the huge impact of the PageRank algorithm [26], many local random walk algorithms have been developed. Kloumann et al. [27] conducted a comprehensive assessment of local community detection algorithms on large graphs. In their study Personal PageRank (PPR) [28] was the clear winner. PPR is able to measure the similarity to a set of vertices instead of the global importance/influence of each vertex by applying a slight modification to PageRank. PageRank can be regarded as a sequence of two step processes that are iterated until convergence: A random walk on the graph followed by (with small probability) a random teleport to any vertex. PPR modifies PageRank in two ways: Only a small number of steps are run (often 4), and any random walker selected to teleport must return to one of the seed vertices. Recent extensions have shown that finding the local community around a vertex can be improved by seeding (using as the teleport set) PPR with the neighborhood graph of that vertex [2] and that PPR can be used to initiate *local* spectral methods with good results [20].

Random walk methods are usually evaluated by power iteration; a series of matrix multiplications requiring the full adjacency matrix to be read into memory. The adjacency matrix of large graphs will not fit in memory. Therefore, distributed computing resources are used (e.g., Hadoop). While distributed systems are continually improving, they are not always available to analysts, require skilled operators and typically have an overhead of several minutes per query.

A major challenge when applying both local and global community detection algorithms to real-world social media networks is performance verification. Testing algorithms on a held-out labeled test set is complicated by the lack of any agreed definition of a community. Much early work makes use of small hand-labeled communities and treats the original researchers’ decisions as gold standards [29–31]. Irrespective of the validity of this process, a single (or small number) of manual labelers can not produce ground-truth for large DSNs. [32] proposed a solution to the verification problem in community detection. They observe that in practice, community detection algorithms detect communities based on the structure of interconnections. However, results are verified by discovering common attributes or functions of vertices within a community. [32] identified 230 real-world networks in which they define ground-truth communities based on vertex attributes. The specific attributes that they use are varied, and some examples include publication venues for academic co-authorship networks, chat group membership within social networks and product categories in co-purchasing networks.

#### Graph processing systems

A complimentary approach to efficient community detection on large graphs is to develop more efficient and robust systems. This is an area of active research within the systems community. General-purpose tools for distributed computation on large scale graphs include Graphlab, Pregel and Surfer [15–17]. Purpose-built distributed graph processing systems offer major advances over the widely used MapReduce framework [33]. This is particularly true for iterative computations, which are common in graph processing and include random walk algorithms. However, distributed graph processing still presents major design, usability and latency challenges. Typically, the run times of algorithms are dominated by communication between machines over the network. Much of the complexity comes from partitioning the graph to minimize network traffic. The general solution to the graph partitioning problem, placing roughly equal numbers of nodes on each machine while minimizing the number of inter-machine edges, is NP-hard and remains unsolved. These concerns have led us and other researchers to buck the overarching trend for increased parallelization on ever larger computing clusters and search for single-machine graph processing solutions. One such solution is Graphci, a single-machine system that offers a powerful and efficient alternative to processing on large graphs [18]. The key idea is to store the graph on disk and optimize input/output (I/O) routines for graph analysis operations. Graphci achieves substantial speed-ups compared to conventional systems, but the repeated disk I/O makes real-time operation impossible. Twitter also use a single-machine recommendation system that serves “Who To Follow (WTF)” recommendations across their entire user base [19]. WTF provides real-time recommendations using random walk methods similar to PPR. This is achieved by loading the entire Twitter graph into memory. Following their design specification of 5 bytes per edge 5 × 30 × 10^9^ = 150 GB of RAM would be required to load the current graph, which is an order of magnitude more than available on a laptop, which serves as our target platform.

#### Graph compression and data structures

The alternative to using large servers, clusters or disk storage for processing large graphs is to compress the whole graph to fit into the memory of a single machine. Graph compression techniques were originally motivated by the desire for single machine processing on the Web Graph. Approaches focus on ways to store the differences between graphs instead of the raw graph. Adler et al. [34] searched for web pages with similar neighborhood graphs and encoded only the differences between edge lists. The seminal work by Boldi et al. [35] ordered web pages lexicographically endowing them with a measure of locality. Similar compression techniques were adapted to social networks by Chierichetti et al. [36]. They replaced the lexical ordering with an ordering based on a single minhash value of the out-edges, but found social networks to be less compressible than the Web (14 bits versus 3 bits per edge). While the aforementioned techniques achieve remarkable compression levels, they pay the price of slower access to the data [19].

Minhashing is a technique for representing large sets with fixed-length signatures that encode an estimate of the similarity between the original sets. When the sets are sub-graphs minhashing can be used for lossy graph compression. The pioneering work on minhashing was by Broder [37] whose implementation dealt with binary vectors. This was extended to counts (integer vectors) by Charikar et al. [38] and later to continuous variables [39]. Efficient algorithms for generating the hashes are discussed by Manasse et al. [40]. Minhashing has been applied to clustering the Web by Haveliwala et al. [41], who considered each web page to be a bag of words and built hashes from the count vectors.

Two important innovations that improve upon minhashing are b-Bit minhashing [42] and Odd Sketches [43]. When designing a minhashing scheme there is a trade off between the size of the signatures and the variance of the similarity estimator. Li et al. [42] show that it is possible to improve on the size-variance trade off by using longer signatures, but only keeping the lowest b-bits of each element (instead of all 32 or 64). Their work delivers large improvements for very similar sets (more than half of the total elements are shared) and for sets that are large relative to the number of elements in the sample space. Mitzenmacher et al. [43] improved upon b-bit minhashing by showing that for approximately identical sets (Jaccard similarities ≈ 1) there was a more optimal estimation scheme.

Locality Sensitive Hashing (LSH) is a technique introduced by Indyk and Motwani [44] for rapidly finding approximate near neighbors in high dimensional space. In the original paper a parameter *ρ* governs the quality of LSH algorithms. A lower value of *ρ* leads to a better algorithm. There is a great deal of work studying the limits on *ρ*. Of particular interest, Motwani et al. [45] use a Fourier analytic argument to provide a tighter lower bound on *ρ*, which was later bettered by O’Donnell et al. [46] who exploited properties of the noise stability of boolean functions. The latest LSH research uses the structure of the data, through data dependent hash functions [47] to get even tighter bounds. As the hash functions are data dependent, unlike earlier work, only static data structures can be addressed.

## Data

In this article we focus on Twitter data because Twitter is the most widely used Digital Social Network (DSN) for academic research and the data is relatively easy to obtain. At the time of writing the Twitter Follower graph consists of roughly one billion vertices (Twitter accounts) and 30 billion edges (Follows).

To collect data we use the Twitter REST API to crawl the network. Every time a new account is crawled we check the number of Followers in the account metadata and if it is greater than 10,000, we download the full Follower list. While 10,000 is an arbitrary number, accounts with more than 10,000 Followers tend to have public profiles (Wikipedia pages or websites), which are required to verify any results.

Our data set is a snapshot of the Twitter graph from December 2015. We found 675,000 accounts with over 10,000 Followers. They are Followed by a total of 1.5 × 10^10^ Followers, of which 7 × 10^8^ are unique. We learn minhash representations of the 675,000 largest accounts using the Following patterns of all 7 × 10^8^ accounts in the dataset. Any queries or results returned in the experimentation section are restricted to the 675,000 hashed accounts.

To show that our method generalizes to other social networks, we also present results using a large Facebook Pages engagement graph containing 450 million vertices (FB accounts) and 700 million edges (Page likes/comments).

It is not possible to crawl the Facebook network in the same way as Twitter and so to collect data from Facebook we matched the Twitter accounts with greater than 10,000 Followers to Facebook Page accounts using a combination of automatic account name matching and manual verification. Facebook Page likes are not available retrospectively, but can be collected through a real-time stream. Having identified the set of accounts on Facebook corresponding to the large Twitter accounts, we used the Facebook API to collect the interaction streams of each page over a period of two years. Due to privacy concerns neither the Twitter nor the Facebook datasets can be made publicly available in their raw forms and so for reproducibility we provide additional results on a public email network dataset [48]. The network is a directed network of email communication from a large European research organization. Each vertex is an employee and they are uniquely labeled by their department (further details and the data are available at http://snap.stanford.edu/data/email-Eu-core.html)

Downloading large quantities of social media data is an involved subject and we include details of how we did this in S1 File for reproducibility.

## Method

In the following, we detail our approach to real-time community detection in large social networks with the restriction that it runs on a single laptop. Our method consists of two main stages: In stage one, we take a set of seed accounts and expand this set to a larger group containing the most related accounts to the seeds. This stage is depicted by the box labeled “Find similar accounts” in Fig 1. Stage one uses a very fast nearest-neighbor search algorithm. In stage two, we embed the results of stage one into a *complete weighted graph* where each vertex is connected to every other vertex. The edge weights are given by the Jaccard similarity of the two accounts they connects. This form of graph is known as an *intersection graph* in the mathematics literature, where it is a well studied object [49–51]. We apply a global community detection algorithm to the intersection graph and visualize the results. Stage two is depicted by the box labeled “Structure and visualize” in Fig 1.

In the remainder of the paper we use the following notation: The *i*^*th*^ user account (or interchangeably, vertex of the network) is denoted by *A*_*i*_, and *N*(*A*_*i*_) gives the set of all accounts directly connected to *A*_*i*_ (the neighbors of *A*_*i*_). The set of accounts for which we want to discover communities in the network are provided by a user into the system and are called “seeds”. They are denoted by *S* = {*A*_1_, *A*_2_, …, *A*_*m*_} while *C* = {*A*_1_, *A*_2_, …, *A*_*n*_} (community) is used for the set of accounts that are returned by stage one of the process.

### Stage 1: Seed expansion

The first stage of the process takes a set of seed accounts as input (provided by the user), orders all other accounts by similarity to the seeds and returns an expanded set of accounts similar to the seed account(s). For this purpose, we require three ingredients:

1. A similarity metric between accounts
2. An efficient system for finding similar accounts
3. A stopping criterion to determine the number of accounts to return

In the following, we detail these three ingredients of our system, which will allow for real-time community detection in large social networks on a standard laptop.

#### Similarity metric

The property of each account that we choose to compare is the neighborhood graph. The neighborhood graph is an attractive feature as it is not controlled by an individual, but by the (approximately) independent actions of large numbers of individuals. The edge generation process in Digital Social Networks (DSNs) is very noisy, producing graphs with many extraneous and missing edges. As an illustrative example, the pop stars Eminem and Rihanna have collaborated on four records and a stadium tour (“Love the Way You Lie” (2010), “The Monster” (2013), “Numb” (2012), and “Love the Way You Lie (Part II)” (2010), the Monster Tour (2014)). Despite this clear association, at the time of writing Eminem is not one of Rihanna’s 40 million Twitter followers. However, Rihanna and Eminem have a Jaccard similarity of 18%, making Rihanna Eminem’s 6^th^ strongest connection. Using the neighborhood graph as the unit of comparison between accounts mitigates against noise associated with the unpredictable actions of individuals. The metric that we use to compare two neighborhood graphs is the *Jaccard similarity*.

The Jaccard similarity is given by

$$\begin{array}{c} J\left(A_{i},A_{j}\right)=\frac{|N\left(A_{i}\right)\cap N\left(A_{j}\right)|}{|N\left(A_{i}\right)\cup N\left(A_{j}\right)|}\;, \end{array}$$ (1)

where *N*(*A*_*i*_) is the set of neighbors of *i*^*th*^ account. The Jaccard similarity has two attractive properties for this task. First, it is a normalized measure providing comparable results for sets that differ in size by orders of magnitude. Second, minhashing can be used to provide an unbiased estimator of the Jaccard similarity that is both time and space efficient.

#### Efficient system to find similar accounts

To efficiently search for accounts that are similar to a set of seeds we represent every account as a minhash signature and use a Locality Sensitive Hashing (LSH) data structure based on the minhash signatures for approximate nearest neighbor search.

#### Computing Jaccard similarities

Computing the Jaccard similarities in Eq (1) is very expensive: Each set of neighbors can have up to 10^8^ members, and calculating intersections is super-linear in the total number of members of the two sets being intersected. Multiple large intersection calculations can not be processed in real-time. There are two alternatives: Either the Jaccard similarities are pre-computed for all possible pairs of vertices, or they are estimated. Using pre-computed values for all *n* = 675,000 Twitter accounts with more than 10,000 Followers would require caching $\frac{1}{2}n\left(n-1\right)\approx 2.5\times 10^{11}$ floating point values requiring approximately 1 TB and exceeding the specifications of a laptop. Therefore, we decide to efficiently estimate the Jaccard similarities using minhashing.

The minhashing compression technique of Broder [52] generates unbiased estimates of the Jaccard similarity in *O*(*K*), where *K* is the number of hash functions in the signature. Theoretical guarantees for minhashing require that min-wise independent permutations of the sets can be efficiently generated. In practice, this is not true and hash functions are used that approximate the minwise independent permutations. This is achieved by first indexing the universe of elements to be hashed. The indices are mapped through a hash function and the value of minimum occupied index for each set is taken as the minhash. Using hash functions to simulate permutations leads to slightly weaker, but still practically useful guarantees [52]. The estimate $\hat{J}\left(A_{i},A_{j}\right)$ of the Jaccard similarity *J*(*A*_*i*_, *A*_*j*_) is attained by exploiting that the probability of a minhash function *h*_*k*_ is equal for two sets *A*_*i*_, *A*_*j*_ is given by the Jaccard coefficient

$$\begin{array}{c} J\left(A_{i},A_{j}\right)=p\left(h_{k}\left(A_{i}\right)=h_{k}\left(A_{j}\right)\right)\;\forall k=1,\ldots ,K\;. \end{array}$$ (2)

Therefore, we obtain an estimate of *J*(*A*_*i*_, *A*_*j*_) by estimating the probability *p*(*h*_*k*_(*A*_*i*_) = *h*_*k*_(*A*_*j*_)). For this, we create a signature vector *H*, which is made of *K* independent hashes *h*_*k*_, *k* = 1, …, *K* and calculate the Monte-Carlo Jaccard estimate $\hat{J}$ as

$$\begin{array}{cc} & \hat{J}\left(A_{i},A_{j}\right)=\frac{I}{K} \end{array}$$ (3)

where we define

$$\begin{array}{cc} & I=\sum_{k=1}^{K}\delta \left(h_{k}\left(A_{i}\right),h_{k}\left(A_{j}\right)\right)\;, \end{array}$$ (4)

$$\delta (h_{k}(A_{i}),h_{k}(A_{j}))=\{\begin{array}{c} 1\;\text{if}\;h_{k}(A_{i})=h_{k}(A_{j})\\ 0\;\text{if}\;h_{k}(A_{i})\neq h_{k}(A_{j}) \end{array}.$$ (5)

As each *h*_*k*_ is independent, *I* ∼ *Bin*(*J*(*A*_*i*_, *A*_*j*_), *K*). The estimator is fully efficient, i.e., the variance is given by the Cramér-Rao lower bound

$$\begin{array}{c} \text{var}\left(\hat{J}\right)=\frac{J(1-J)}{K}\;, \end{array}$$ (6)

where we have dropped the Jaccard arguments for brevity. Eq (6) shows that Jaccard coefficients can be approximated to arbitrary precision using minhash signatures with an estimation error whose variance scales as *O*(1/*K*).

#### Memory and space improvements of minhashing

We use all 700 million Twitter accounts are used to compute the minhash signatures. However, the memory requirement of minhash signatures is only *Kn* integers, where *K* is the number of hash functions and *n* is the number of considered Twitter accounts. Therefore, it fits into the RAM of a laptop: For *K* = 1000 independent hash functions and the *n* = 675,000 largest Twitter accounts, only ≈ 4*GB* are required. In comparison to calculating Jaccard similarities of the largest 675,000 Twitter accounts with ≈ 4 × 10^10^ neighbors minhashing reduces expected processing times by a factor of 10,000, and storage space by a factor of 1,000. Note that our method allows to add new accounts quickly by simply calculating one additional minhash signature without needing to add the pairwise similarity to all other accounts.

**Algorithm 1** Minhash signature generation

**Require:** *M* ← number of Accounts

**Require:** *K* ← size of signature

**Require:** *N*(*Account*) ← All neighbors

1. $T\in N^{M\times K}\leftarrow \infty$           ⊳ Initialise signature matrix to ∞

2. index ← 1

3. **for all** Accounts **do**

4.  $P\leftarrow \text{permute}\left(\text{index}\right)\in N^{1\times K}$   ⊳ Permute the Account index *K* times

5.  **for all** N(Account) **do**

6.   *T* [*i*]←min(*T* [*i*], *P*) ⊳ Compute the element-wise minimum of the signature

7.  **end for**

8.  index = index + 1

9. **end for**

10. **return** *T*                  ⊳ Return matrix of signatures

#### Efficient generation of minhash signatures

Minhash signatures allow for rapid estimation of the Jaccard similarities. However, care must be taken when implementing minhash generation. Calculation of the signatures is expensive: Algorithm 1 requires *O*(*NEK*) computations, where *N* is the number of neighbors, *E* is the average out-degree of each neighbor and *K* is the length of the signature (i.e., the number of independent hash functions for estimating the Jaccard similarity). For our Twitter data these values are *N* = 7 × 10^8^, *E* = 10, *K* = 1,000. A naive implementation can run for several days. We have an efficient implementation that takes one hour allowing signatures to be regenerated overnight without affecting operational use (see S1 File for more details).

#### Locality Sensitive Hashing (LSH)

Calculating Jaccard similarities based on minhash signatures instead of full adjacency lists provides tremendous benefits in both space and time complexity. However, finding near neighbors of the input seeds is an onerous task. For a set of 100 seeds and our Twitter data set, nearly 70 million minhash signature comparisons would need to be performed, which dominates the run time. Locality Sensitive Hashing (LSH) is an efficient system for finding approximate near neighbors [44].

**Fig 2:**
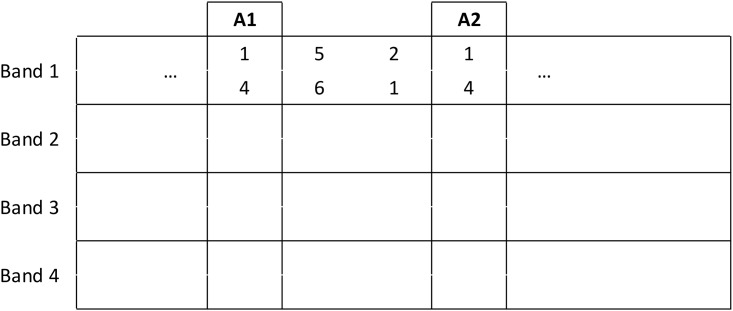
Illustration of LSH applied to minhash signatures. Each row represents a signature. The signatures have been banded up, so that each band contains two hashes. Accounts A1 and A2 will be grouped as similar candidates since they have identical signatures in Band 1.

LSH works by partitioning the data space. Any two points that fall inside the same partition are regarded as similar. Multiple independent partitions are considered, which are invoked by a set of hash functions. LSH has an elegant formulation when combined with minhash signatures for near neighbor queries in Jaccard space, which is illustrated in Fig 2. The minhash signatures are divided into bands containing fixed numbers of hash values. LSH exploits that similar minhash signatures are likely to have identical bands. An LSH table can then be constructed that points from each account to all accounts that have at least one identical minhash band. In Fig 2, accounts A1 and A2 have an identical Band 1 and so will be grouped together. We apply LSH to every input seed independently to find all candidates that are ‘near’ to at least one seed.

In our implementation, we use 500 bands, each containing two hashes. For every query account the LSH table is used to lookup similar (high Jaccard coefficient) accounts. Each lookup is O(1) and so the total runtime is O(S) where S is the number of seeds in the query. The results are stored as a set (the candidates set) so that any duplicates resulting from separate queries are removed. Table 2 shows typical system runtimes for three scenarios: a naive implementation, using just minhashing and using LSH and minhashing. As most accounts share no neighbors, the LSH step dramatically reduces the number of candidate accounts and the algorithm runtime by a factor of roughly 100. LSH is essential for the real-time capability of our system.

**Table 2:** Typical runtimes and space requirements for systems performing local community detection on the Twitter Follower network of 700 million vertices and 20 billion edges and producing 100 vertex output communities.

System	Typical runtime (s)	Space requirement (GB)
Naive edge list	8,000	240
Minhash signatures	1	4
LSH with minhash	0.25	5

#### Sorting similarities

LSH produces a set of candidate accounts that are related to at least one of the input seeds. In general, we do not want every candidate returned by LSH. Therefore, we select the subset of candidates that are most associated with the seed set defined by the user.

We experimented with two sequential ranking schemes: Minhash Similarity (MS) and Agglomerative Clustering (AC). In both cases accounts are ranked based on their distance to the center of the seeds **X**. The *j*^th^ component of the distance is given by

$$X_{j}=\frac{1}{n}\sum_{i=1}^{n}D\left(A_{j},S_{i}\right)\;,\;j=1,\ldots ,M.$$ (7)

**Fig 3:**
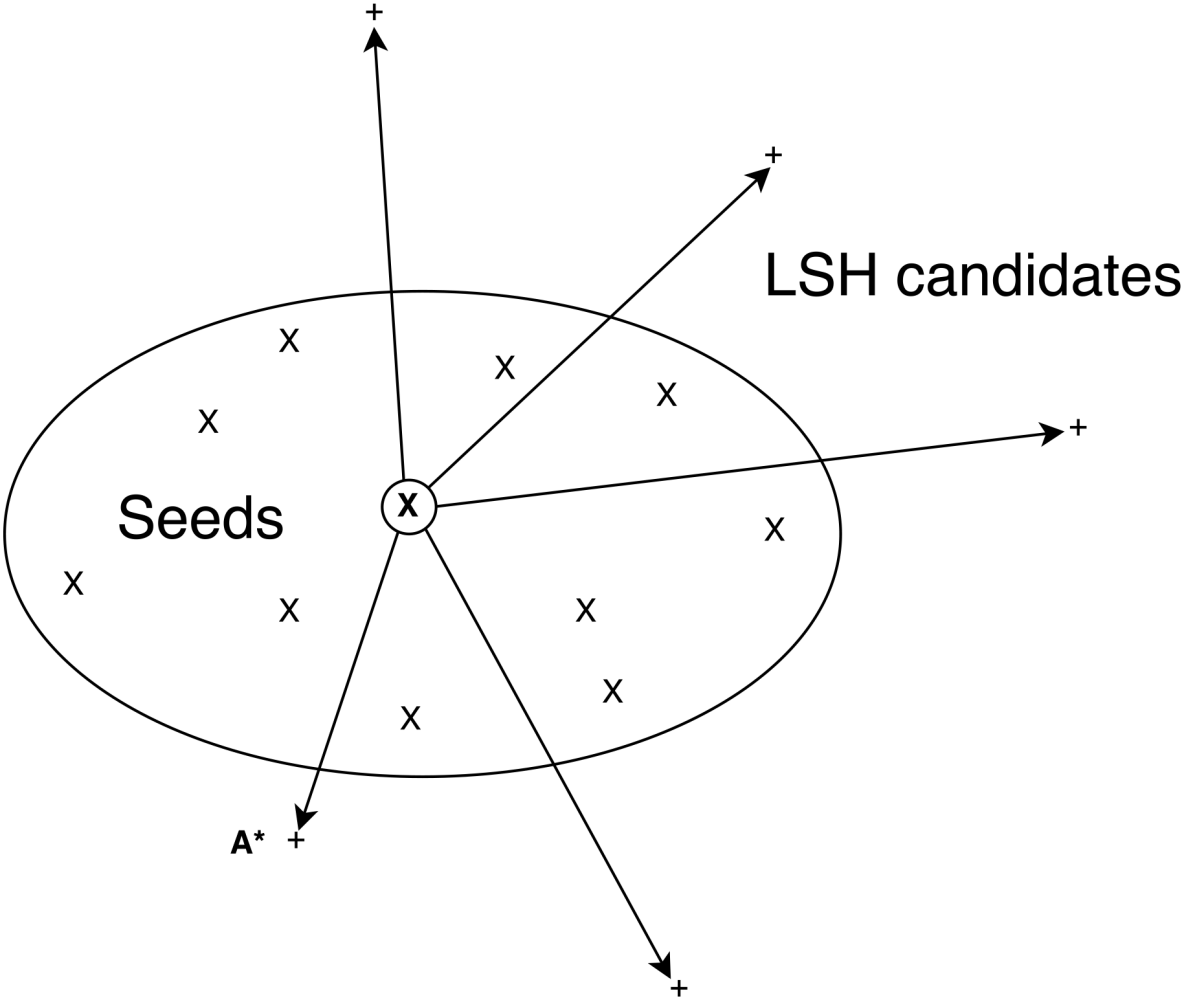
Sorting similarities of LSH candidates. The diagram shows a set of seed accounts (X) bounded by an ellipse in Jaccard space. Outside of the ellipse are a set of LSH candidate accounts (+). At each iteration the candidate account (A*) closest (according to the Jaccard distance) to the center (**X**) of the seeds is added to the list of returned values.

whereD=1−J (8)

and *M* is the number of accounts in the dataset. At each step AC and MS augment the results set *C* with A*, the closest account to **X** with A* ∉ *C*. However, MS uses a constant value of **X** based on the *n* input seeds while AC updates **X** after each step. Fig 3 illustrates the ranking process. A set of seeds are shown with their center ringed. A* denotes the closest account to the center.

**Algorithm 2** Agglomerative Clustering Algorithm (AC)

**Require:** Initial seeds S

1. Define candidate members $\bar{A}=LSH\left(S\right)$

2. *C*_0_ = *S*

3. **repeat**

4.  Compute community centre $X(\bar{A},C_{t})$, see (7)

5.  Select next Account: A* = argmin_*A*_*i*_ ∉ *C*_ **X**(*A*_*i*_, *C*_*t*_)

6.  Grow community: *C*_*t*+1_ ← *C*_*t*_ ∪ A*, $\bar{A}\leftarrow \bar{A}\backslash A^{*}$

7. **until** Stopping criterion is met

At each iteration of Algorithm 2 *C* and **X** are updated by first setting *C* = *S* and then adding the closest account given by

$$A_{t}^{*}=\arg\min_{A_{j}\notin C_{t}}\sum_{i=1}^{n}D\left(A_{j},{\left(C_{t}\right)}_{i}\right)$$ (9)

leading to

$$\begin{array}{c} C_{t+1}=C_{t}\cup A^{*}. \end{array}$$

The new center **X**_**n**+**1**_ is most efficiently calculated using the recursive online update equation

$$\begin{array}{c} X_{t+1}\left(A,C_{t+1}\right)=\frac{nX\left(A,C_{t}\right)+D\left(A^{*},C_{t}\right)}{n+1}, \end{array}$$ (10)

where *n* is the size of *C*_*t*_ and the scalar operations are broadcast over each element of **X**(**A**, **C**_**t**_).

#### Stopping criterion

Both AC and MS are sequential processes and will return every candidate account unless a stopping criterion is applied. Many stopping criteria have been used to terminate seed expansion processes. The simplest method is to terminate after a fixed number of inclusions. Alternative methods use local maxima in modularity [53] and conductance [54]. In the context of our application, we choose a different criterion: *coverage*. We refer to the number of unique neighbors of a set of accounts as the *coverage* of that set.

The reason for choosing coverage as the stopping criterion is driven by one application of our work: To help define an optimal set of influencers to endorse a brand. In this context, we want to answer questions like: “What is the smallest set of athletes that have influence on over half of the users of Twitter?”. This is captured by the coverage. However, computing the coverage is combinatorial and requires calculating large numbers of unions over very large sets. However, it can be efficiently approximated using minhash signatures. We exploit two properties of minhash signatures to do this: The unbiased Jaccard estimate through Eq 3 and the property that the minhash signature of the union of two sets is the element-wise minimum of their respective minhash signatures. Therefore, the properties of minhash signatures allow us to efficiently compute the coverage and use it as a stopping criterion to rank LSH candidates without losing real-time performance.

#### Efficient coverage computation

The coverage *y* is given by

$$\begin{array}{c} y=\left|\overset{n}{\underset{i=1}{⋃}}N\left(A_{i}\right)\right|, \end{array}$$ (11)

the number of unique neighbors of the output vertices. Every time a new account *A* is added we need to calculate |*N*(*C*) ∪ *N*(*A*)| to update the coverage. This is a large union operation and expensive to perform on each addition. Eq (12) allows us to rephrase this expensive computation using the Jaccard coefficient (available cheaply via the minhash signatures), which we subsequently use for a real-time iterative algorithm. Using Eq (1) and the inclusion-exclusion principle

$$\begin{array}{c} |N(A\cup C)|=|N(A)\cup N(C)|=\frac{\left|N(A)|+|N(C)\right|}{1+J(A,C)}. \end{array}$$ (12)

As minhash signatures are composable sketches, community *C* = ⋃_*i*_ *A*_*i*_ can be represented by a minhash signature

$$H(C)=\{h_{1}\left(C\right),h_{2}\left(C\right),\ldots ,h_{K}\left(C\right)\}$$ (13)

$$\text{where}\;h_{k}\left(C\right)=\min_{j}\left(h_{k}\left(A_{j}\right)\right),\;k=1,\ldots ,K.$$ (14)

We use Eq (12) to update the unique neighbor count, once the next account to add to the community A* is determined according to Eq (9). Then, the neighbor count of the new (augmented) community is

$$\begin{array}{c} |N\left(C_{t+1}\right)|=\frac{|N\left(C_{t}\right)|+|N\left(A^{*}\right)|}{1+J(C_{t},A^{*})}. \end{array}$$ (15)

The right hand side of Eq (15) contains three terms: |*N*(*C*_*t*_)| is what we started with, |*N*(A*)| is the neighbor count of the newly added account A* and *J*(*C*_*t*_, A*) is a Jaccard calculation between the previous community *C*_*t*_ and the new account A*. The minhash signature of a community is obtained via Eq (13). Hence, we can calculate the coverage with negligible additional computational overhead.

The process is then stopped after *T* iterations where *T* is the smallest integer s.t.

$$\begin{array}{c} |N\left(C_{T}\right)|>n_{c} \end{array}$$ (16)

with *n*_*c*_ the required coverage, which is specified on a task by task basis.

### Stage 2: Community detection and visualization

Stage 1 expanded the seed accounts used to query the system to include a larger set of similar accounts. Starting from the seeds, this was achieved by (1) finding a large group of candidates using LSH that were related to any of the seeds and (2) filtering down to the accounts most closely associated to the whole seed set.

**Fig 4:**
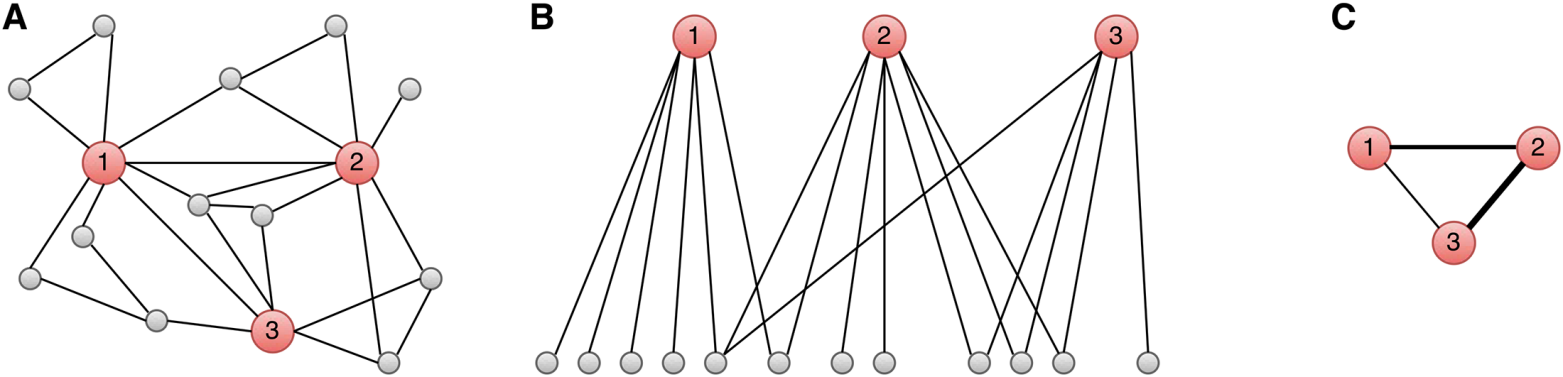
Visualizing the intersection graph generation. Interesting vertices are depicted as larger red nodes, and the neighbors as smaller, more numerous gray nodes. **A** shows a complete social network. **B** depicts the overlapping bipartite neighborhood graphs of the three interesting vertices in **A**. **C** summarizes the social network in **A** by an inferred network using the Jaccard similarity measure of the set of neighboring vertices as edge weights. Vertices connected by high weights are more likely to be in the same community.

In Stage 2, the vertices returned by Stage 1 are used to construct an intersection graph where the edge weights are given by the Jaccard co-efficients of the neighborhood graphs. Fig 4 depicts the process of transforming the original unweighted graph into an intersection graph. **A** shows the vertices (red larger circles) that are returned by stage one and their Followers (gray smaller circles). **B** shows a bipartite graph in which each red vertex is connected to its Followers. The specific representation used is a minhash signature that captures the Jaccard similarity between sets of Followers. The Jaccard similarities obtained through minhash signatures are interpreted as edge weights in **C**, thereby producing a complete intersection graph in which every red vertex is connected to every other by a weighted edge. The edge weights are calculated for all pairwise associations from the minhash signatures through Eq 3. This process effectively embeds the original graph in a metric Jaccard space [37]. Community detection is run on the weighted graph.

The final element of the process is to visualize the community structure and association strengths in the region of the input seeds. We experimented with several global community detection algorithms. These included INFOMAP, Label Propagation, Betweenness Centrality, Leading Eigenvector, Spinglass and Modularity Maximization [13, 55–58]. The Jaccard similarity graph is a complete weighted graph and most community detection algorithms are designed for binary sparse graphs. As a result, all methods with the exception of label propagation and WALKTRAP were too slow for our use case. Label Propagation had a tendency to select a single giant cluster, thus adding no useful information. Therefore, we chose WALKTRAP for community visualization.

### Time complexity

To analyze the complexity of the end-to-end process we use |*S*| seed accounts, |*C*| output accounts, minhash signatures of size *K* and *C*_*a*_ LSH candidates. Here we first analyze the simplest case of MS with a fixed number of (|*C*|) output values and then discuss how this is modified by applying AC and a dynamic stopping criteria.

**Fig 5:**
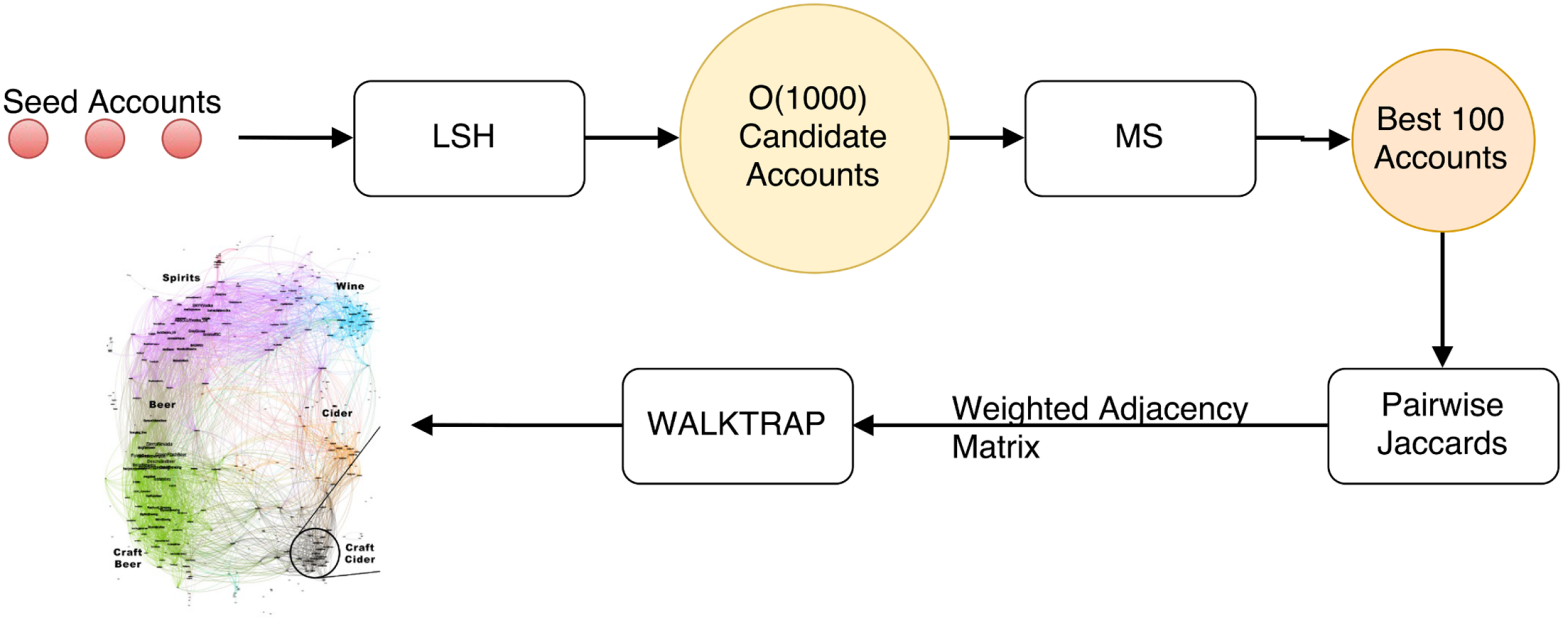
The full process diagram. A set of seeds is queried using LSH and Minhash Similarity. The weighted adjacency matrix for the top 100 results is estimated using minhash signatures. The WALKTRAP community detection algorithm is applied to the weighted adjacency matrix, and the results are visualized.

The end to end process diagram is visualized in Fig 5. The first step is to run |*S*| LSH queries with *O*(|*S*|) runtime. The results are returned as Python sets and finding the union is *O*(∑_*s*_ *C*_*s*_) where *C*_*s*_ is the number of candidates associated only with seed *s* (runtimes are implementation dependent and for python data structures are given in https://wiki.python.org/moin/TimeComplexity). For each candidate we calculate the average minhash similarity between the candidate and all of the seeds, which is *O*(*K*|*S*|*C*_*a*_). The values are sorted in *O*(*C*_*a*_ log *C*_*a*_) and the top |*C*| are retained as the output vertices. Finding all pairwise Jaccard similarities is *O*(|*C*|^2^ *K*). The reported runtimes of Walktrap for a graph *G*(*V*, *E*) are *O*(|*E*||*V*|^2^) [9]. We apply Walktrap to the complete Jaccard graph, which has *V* = *C* and |*E*| = |*C*|^2^/2 leading to a runtime of *O*(|*C*|^3^). Putting this all together we have a complexity of

$$\begin{array}{c} O(|S|+\sum_{s}C_{s}+K|S|C_{a}+C_{a}\log C_{a}+|C{|}^{2}K+|C{|}^{3}) \end{array}$$

Many of these values are user dependent, typical orders of magnitude for the Twitter data are |*S*| = 10, |*C*| = 100, *C*_*a*_ = 1000, *K* = 1000, *C*_*s*_ = 100. In which case the runtime reduces to

$$\begin{array}{c} O(|C{|}^{2}K+|C{|}^{3}). \end{array}$$

For very large graphs the only values that change are *C*_*a*_ and *C*_*s*_. For *C*_*a*_ > *C*_*s*_ >> *C* the runtime becomes

$$\begin{array}{c} O(\sum_{s}C_{s}+K|S|C_{a}+C_{a}\log C_{a}). \end{array}$$

Applying AC instead of MS introduces additional complexity. In Algorithm 2 there are three stages that are repeated until the stopping criteria is met: (1) Recomputing the center, (2) Finding the nearest account to the center (3) augmenting the result set. Calculating the distance to the center is *O*(*K*|*S*|*C*_*a*_) for the first iteration and then applying Eq (10) is *O*(*C*_*a*_) for each subsequent iteration. Augmenting the result set is *O*(1). This leads to an AC time complexity of

$$\begin{array}{c} O(|S|+\sum_{s}C_{s}+K|S|C_{a}+C_{a}\log C_{a}+|C{|}^{2}K+|C{|}^{3}+|C|C_{a}) \end{array}$$

Finally adding a check of the stopping criteria requires evaluating Eq 15 at each iteration. This is dominated by the Jaccard calculation in the denominator *J*(*C*_*t*_, A*). For MS this introduces an additional *O*(|*C*|*K*) term. However for AC we are calculating *D*(*C*_*t*_, A*) = 1 − *D*(*C*_*t*_, A*) in Eq 10 anyway and so there is no additional time complexity.

### Space complexity

The space complexity is dominated by the need to read the LSH table and the minhash table into memory. The minhash table has *O*(*NK*) space complexity where *N* is the number of accounts to be hashed. In the worst case every account hashes to every other accounts and the LSH table has *O*(*N*^2^) space complexity for a complete graph. For the sparse complex networks that we are interested in this reduces to *O*(*N*) as each vertex only hashes to members of the community that contains it and community size does not scale with the size of the network [59]. This leads to a space complexity of *O*(*NK*).

## Ground-truth communities

Generally, community detection algorithms are based on the structure of the graph [60]. To provide a quantitative assessment of our method we require ground-truth labeled communities. However, no ground truth exists for the size of social networks we consider in this article. Therefore, in the following, we will provide a methodology for generating such a ground truth. The methodology itself must be verified, and we provide an extensive evaluation of the quality of the derived ground-truth based on the *four axioms for good community structures* [32]:

1. Compactness
2. Dense interconnectivity
3. Good separation from the rest of the network
4. Internal homogeneity

However, while communities are detected using these properties, verification typically requires associating each vertex with some functional attributes, e.g., fans of Arsenal football club or Python programmers, and showing that the discovered communities group attributes together [32]. The practice of relating community membership with personal attributes is justified by the *homophily principle* of social networks [4], which states that people with similar attributes are more likely to be connected.

We reverse the process of verification by generating ground truth from personal attributes. To generate attributes we match Twitter accounts with Wikipedia pages and associate Wikipedia tags with each Twitter account. Wikipedia tags give hierarchical functions like ‘football:sportsperson:sport’ and ‘pop:musician:music’. It is not possible to match every Twitter account, and our matching process discovered 127 tags that occur more than 100 times in the data. Many of them were too vague to be useful such as ‘news:media’ or ‘Product Brand:Brands’. We selected 16 tags that had relatively high frequencies in the data set and evaluated 7 metrics for each that are related to the four axioms. Separability and conductance measure how well-separated a community is from the rest of the graph. Density and size measure the compactness and density. Cohesiveness, clustering and conductance ratio measure how internally homogeneous a community is. The mathematical formulation of these metrics and details of how they were calculated is provided in the supplementary material.

**Fig 6:**
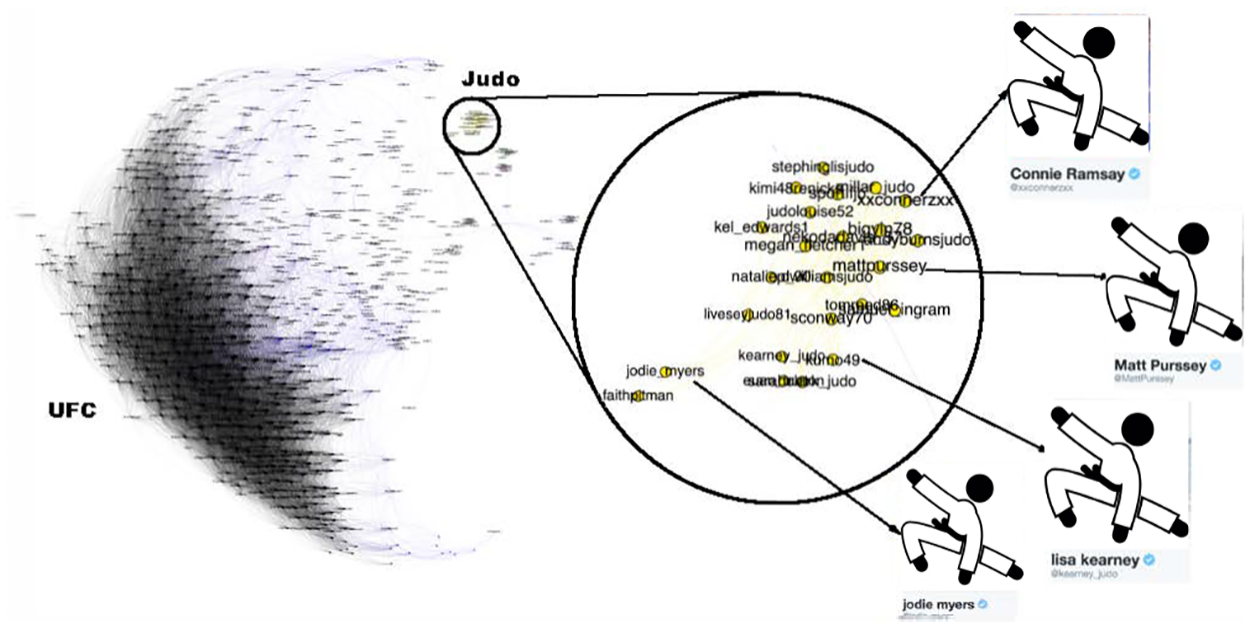
Mixed martial arts (MMA) community. The MMA community is relatively homogeneous and densely interconnected with high clustering and good separability from the rest of the network. The only disconnected region is the yellow region, which has been magnified to show that it is made up of Olympic judo competitors. This community is well detected by all methods.

**Fig 7:**
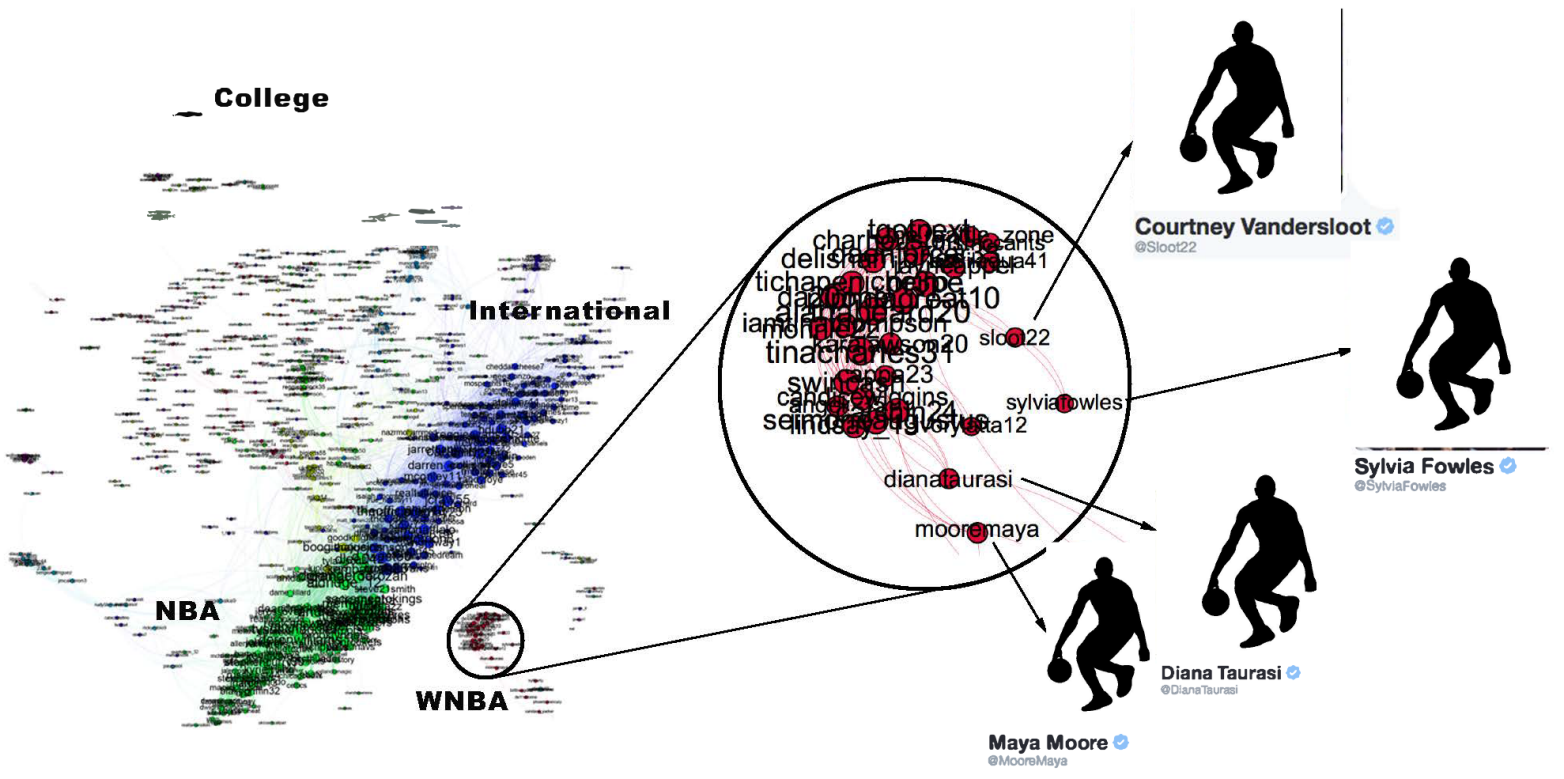
Basketball community. The basketball community has attributes similar to the baseball and American football communities: All are densely connected and well separated from the rest of the network. The individual team structure is not apparent in the graph. Instead the two large clusters show teams from the Eastern and Western Conferences. The small peripheral clusters are mostly major college teams. We have magnified an area showing players of the Womens National Basketball Association.

**Fig 8:**
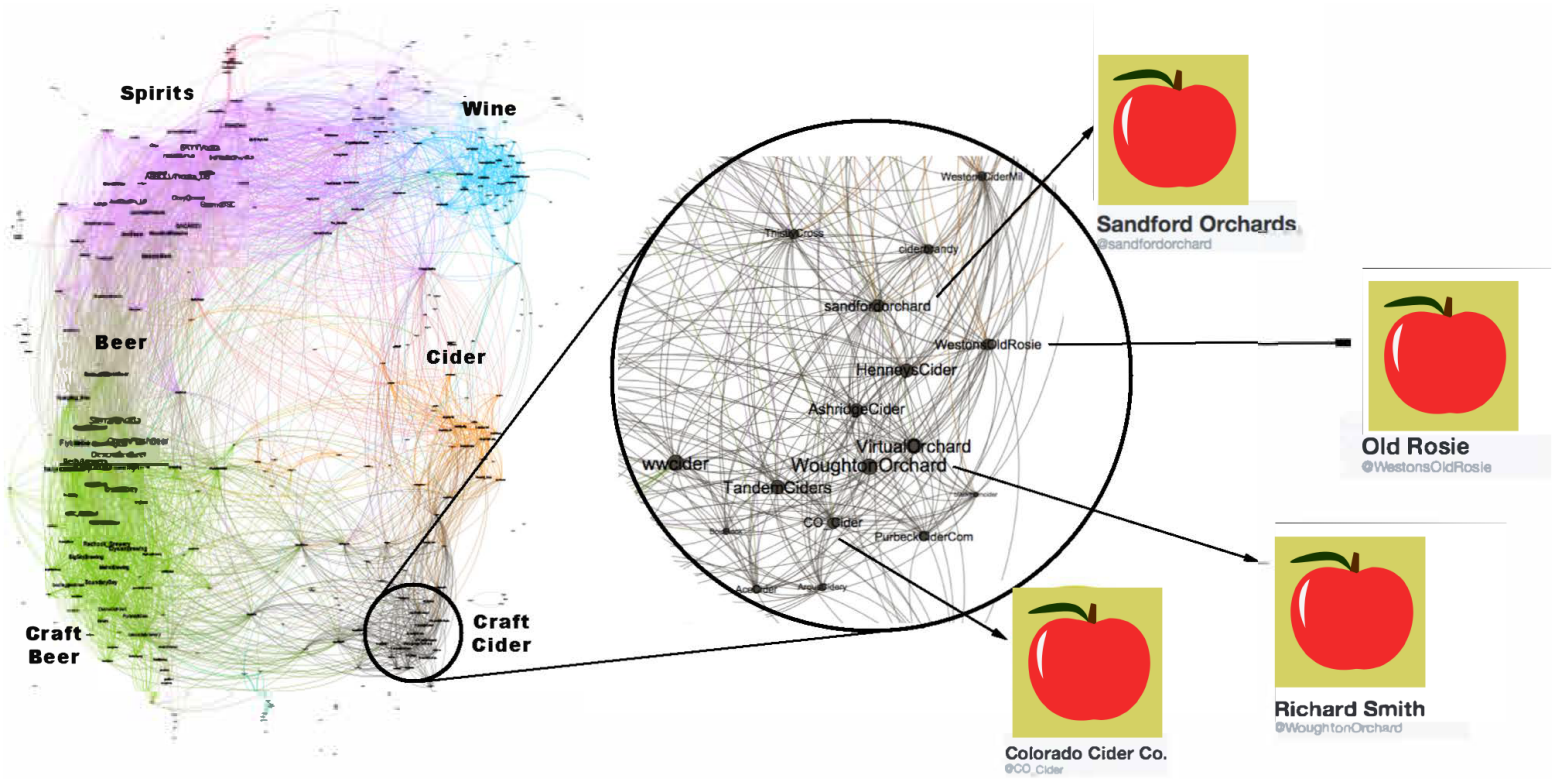
Alcohol community. This is a low-density community with poor clustering. It is divided into broad classes of drinks such as beer, spirits and wine. We have magnified an area of the cider sub-community.

**Fig 9:**
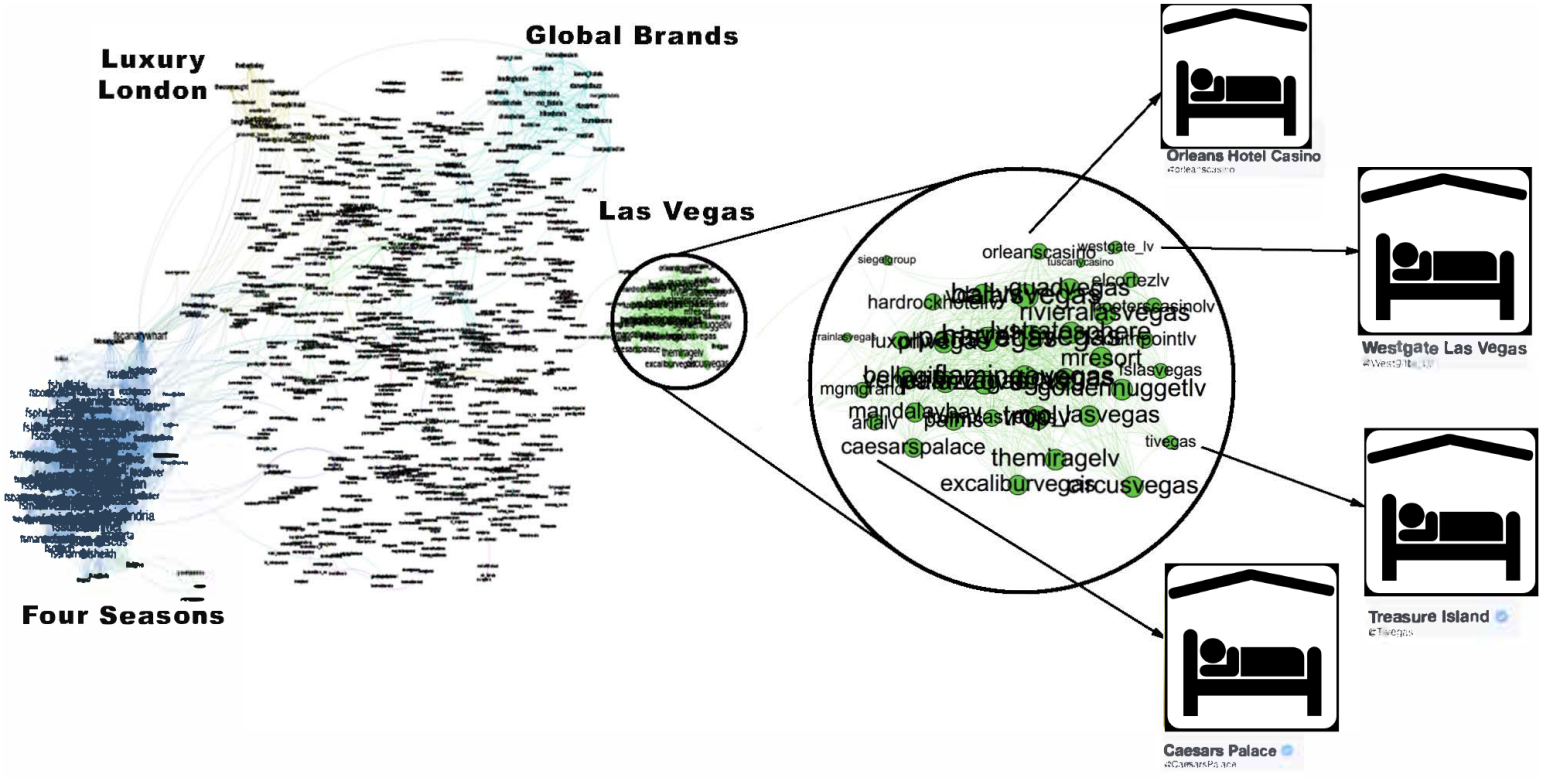
Hotels network. The hotels community has low conductance indicating that it is not well separated from the rest of the network. It also has high cohesiveness indicating it contains components that appear to be the true modular units. The two clearly visible subcomponents are the Four Seasons brand in blue to the left and the hotels of Las Vegas, which is magnified.

The corresponding results are shown in Table 3. Table 3 is sorted by density, and the bold rows are visualized in Figs 6, 7, 8 and 9. The density is the most important factor to distinguish good from bad communities, varying by two orders of magnitude across the data. This is followed by how well separated (separability) the community is from the rest of the network, which is inversely correlated with conductance by design (see S1 File). High clustering is also a useful indicator of community goodness for the best communities, but it is less useful for separating communities that consist of many sub-units (e.g., Team Sports) from very bad communities (e.g., Food and Drink). Cohesiveness is generally not useful as most communities contain at least one well separated sub-unit.

**Table 3:** Properties of ground-truth communities sorted by edge density. CR stands for Conductance Ratio. High values of clustering, density and separability and low values of CR, conductance and Cohesiveness indicate good communities.

Community	Size	Clustering	Cohesiveness	Conductance	CR	Density	Separability
**Mixed Martial Arts**	**751**	**6.49E-02**	**4.29E-01**	**5.10E-01**	**1.19**	**3.06E-02**	**4.80E-01**
**Adult Actors**	352	7.20E-02	1.29E-01	7.70E-01	5.98	2.94E-02	1.50E-01
**Cycling**	371	6.43E-02	4.51E-01	7.04E-01	1.56	2.50E-02	2.11E-01
**Baseball**	616	3.64E-02	1.49E-01	7.87E-01	5.29	1.63E-02	1.35E-01
**Basketball**	**786**	**3.84E-02**	**3.30E-01**	**7.71E-01**	**2.34**	**1.60E-02**	**1.48E-01**
**American Football**	1295	2.24E-02	3.82E-01	7.40E-01	1.94	9.33E-03	1.75E-01
**Athletics**	530	3.48E-02	4.13E-01	8.47E-01	2.05	8.21E-03	9.01E-02
**Hotel Brand**	**836**	**2.20E-02**	**4.53E-01**	**8.37E-01**	**1.85**	**6.16E-03**	**9.71E-02**
**Airline**	363	2.30E-02	4.41E-01	9.46E-01	2.15	4.35E-03	2.84E-02
**Cosmetics**	332	3.34E-02	4.87E-01	9.56E-01	1.96	3.55E-03	2.32E-02
**Football**	4111	3.69E-02	3.95E-01	7.07E-01	1.79	2.93E-03	2.07E-01
**Alcohol**	**388**	**1.72E-02**	**2.34E-01**	**9.52E-01**	**4.06**	**2.66E-03**	**2.53E-02**
**Travel**	2038	1.27E-02	4.25E-01	8.29E-01	1.95	2.50E-03	1.03E-01
**Model**	2096	2.62E-02	4.04E-01	9.01E-01	2.23	1.90E-03	5.50E-02
**Electronics**	689	1.40E-02	4.38E-01	9.75E-01	2.23	8.78E-04	1.30E-03
**Food and Drink**	2974	1.76E-02	4.57E-01	9.06E-01	1.98	7.69E-04	5.18E-02

To establish a clearer view of the density and homogeneity of the ground-truth we visualize the communities using network diagrams and dendrograms. *Network diagrams* are generated in Gephi [61]. The layout uses the Force Atlas 2 algorithm. Colors indicate clusters generated using Gephi’s modularity optimization routine. The node (and label) sizes indicate the weighted degree of each node and are scaled to be between 5 and 20 pixels. The network diagrams reveal any substructure present within the ground-truth. They contain too much information to easily see the individual accounts. Therefore, we magnify small subregions and display Twitter profile images for accounts within them. A weakness of the network diagrams is that different edge weights are hard to perceive.

**Fig 10:**
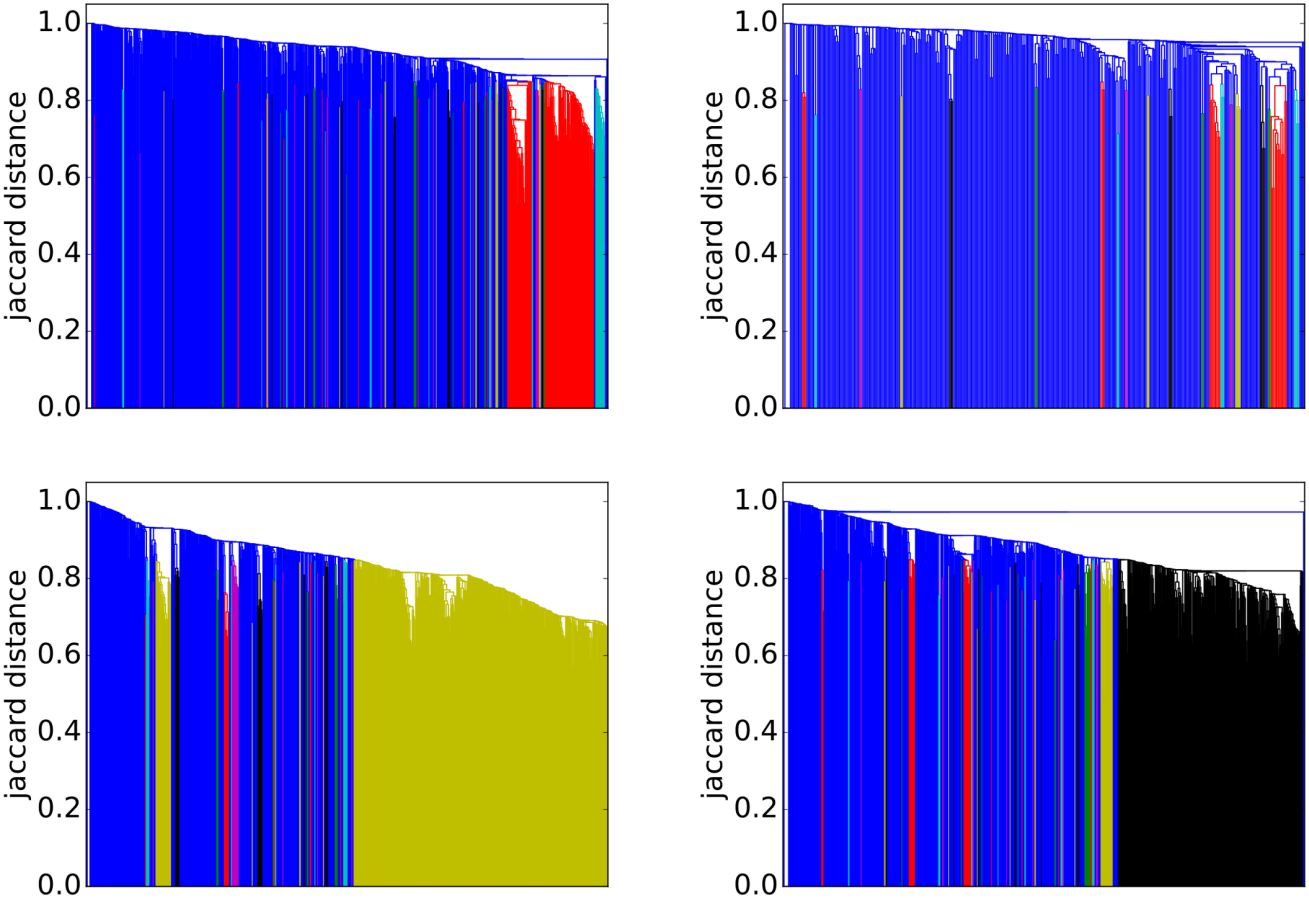
Dendrograms showing the strength of interconnection within communities. The vertical axes show the Jaccard distances. Blue areas are weakly connected. In each colored region, no two nodes are separated by a Jaccard distance greater than 0.85. The dendrograms are agglomerative: All accounts with a Jaccard distance less than the *y*-value are fused together into a super-node. The fusing process is sequential and the *x*-axis indicates the order of fusing with the first nodes to agglomerate at the right. The bottom-right subfigure shows team sports (here: Basketball). There are any highly connected sub-groups. The Bottom-left subfigure shows the most clearly defined communities (here: Mixed Martial Arts) containing sub-communities mostly due to nationality. The top-right subfigure shows industrial groups (here: Alcohol) with limited interactions. The top-left subfigure shows industrial groups (here: Hotels) with small highly connected groups due to sub-brands.

To provide a visual representation of the general strength of interaction we generated dendrograms (Fig 10) for each ground-truth community. *Dendrograms* are agglomerative: All accounts with a Jaccard distance less than the *y*-value are fused together into a super-node. Any subgroups containing more than 10 nodes with no two nodes separated by a Jaccard distance greater than 0.85 have been colored to indicate sub-communities.

Fig 6 shows the Mixed Martial Arts (MMA) community. From Table 3 we see that this community is densely connected, strongly clustered and very well separated from the rest of the network. The gold region in the bottom-right subfigure of Fig 10 is a massive cluster where the distance between any two nodes is less than 0.8. It depicts MMA fighters, mostly fighting in the Ultimate Fighting Championship (UFC). There is a single well-separated sub-community, which is magnified in Fig 6 showing Olympic judo fighters. MMA is the *best* community in our study. The Cycling, Adult Actor and Athletics communities are similar in structure to MMA (see Table 3).

Fig 7 shows that the basketball community (largely NBA players) exhibits two large communities (the two NBA conferences). The individual team structure within the divisions is apparent from the fine banding in the bottom-right subfigure of Fig 10 where many well-connected sub-clusters, each with a distance of less than 0.85 between all pairs of nodes, are visible. We have magnified a small disconnected region of Fig 7, which shows players of the Women’s National Basketball Association (WNBA). Other team sports (Baseball, football and American football) exhibit similar structural properties.

Fig 8 shows that the alcohol industry is split into four major groups representing the different classes of alcoholic drinks (wine, beer, cider and spirits). We have magnified a region of the network that contains mostly English *craft* ciders. The top-right subfigure of Fig 10 shows that the alcohol network is mostly poorly connected with only two colored regions indicating well connected sub-communities. From Table 3 it can be seen that the alcohol network exhibits a low link density and separability, indicating that the community lacks distinction from the rest of the network. This is a consistent pattern for other communities drawn from industrial segmentations (Food and Drink, Electronics, Model).

Fig 9 shows an example of the final group of ground-truth communities: industrial groups with prominent sub-communities. In this case the major sub-communities are the Four Seasons Hotel group and hotels located in Las Vegas (magnified). The top-left subfigure of Fig 10 shows that while the hotel network is generally poorly connected, there are sizable highly interconnected sub-communities. Table 3 shows that the hotel community exhibits low clustering (most accounts are disconnected), high cohesiveness (there are well connected sub-groups) and a low conductance ratio. The travel, airlines and cosmetics communities all share these traits (see Table 3).

In summary, we identified four types of ground-truth communities and evaluated their quality based on the four axioms. We found that the community types differ greatly in quality. The group containing mixed martial arts, cycling, athletics and adult actors satisfies the four axioms and form a good set of ground-truth for algorithm evaluation. The group comprising team sports (American football, baseball, basketball and football) satisfy three of the four axioms (they are not homogeneous). The remaining two community types only contain sub-groups that satisfy any of the axioms.

## Experimental evaluation

Our approach to real-time community detection relies on two approximations: minhashing for rapid Jaccard estimation and locality sensitive hashing to provide a fast query mechanism (based on minhashing). We assess the effect of these approximations, and demonstrate the quality of our results in three experiments:

1. We measure the sensitivity of the Jaccard similarity estimates with respect to the number of hash functions used to generate the signatures. This will justify the use of the minhash approximation for computing approximate Jaccard similarities.
2. We compare the runtime and recall of our process on ground-truth communities against the Personal Page Rank (PPR) algorithm (state of the art) on a laptop.
3. We visualize detected communities and demonstrate that association maps for social networks using minhashing and LSH produce intuitively interpretable maps of the Twitter and Facebook graphs in real-time on a laptop.

### Experiment 1: Assessing the quality of Jaccard estimates

**Fig 11:**
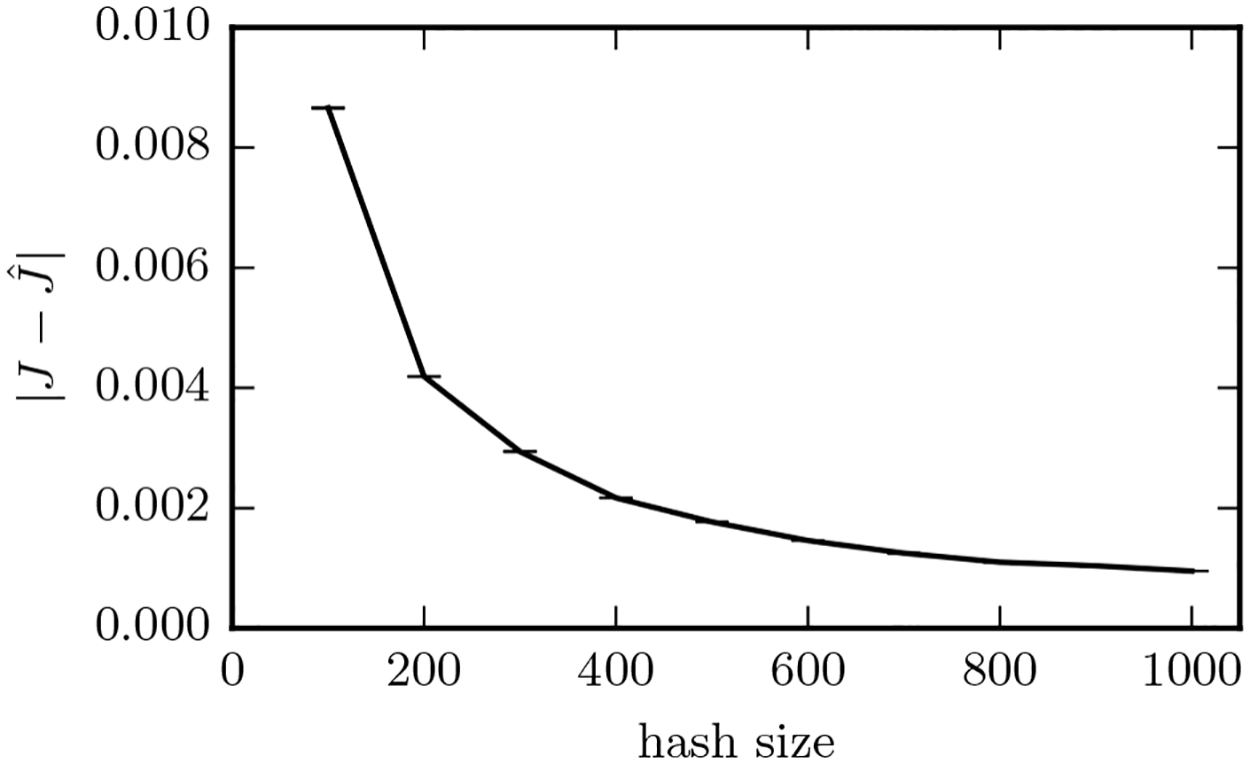
Expected error from Jaccard estimation using minhash signatures as a function of the number of the hashes used in the signature. The error bars show twice the standard error using 400,000 data points.

We empirically evaluate the minhash estimation error using a sample of 400,000 similarities taken from the 250 billion pairwise relationships between the Twitter accounts in our study. We compare estimates using Eq (3) to exact Jaccards obtained by exhaustive calculations on the full sets using Eq (1). Fig 11 shows the estimation error (L1 norm) as a function of the number of hashes comprising the minhash signature. Standard error bars are just visible up until 400 hashes. The graph shows an expected error in the Jaccard of just 0.001 at 1,000 hashes. The high degree of accuracy and diminishing improvements at this point led us to select a signature length of *K* = 1,000. This value provides an appropriate balance between accuracy and performance (both runtime and memory scale linearly with *K*).

In a practical application, we are interested in finding accounts that are similar to seed accounts. We then rank other accounts based on similarity. Local community detection algorithms add accounts in similarity order. Therefore, approximating the true ordering is an important property.

In a concrete case, we looked at the Nike Twitter account as a seed and computed the Jaccard similarities to other Twitter accounts using a) ground truth (which is expensive to compute) and b) our proposed estimation using minhash signature of length *K* = 1,000. A top-ten ranked list of Jaccard similarities is given in Table 4 for the Nike Twitter account (based on the true Jaccard). Possible matches include sports people, musicians, actors, politicians, educational institutions, media platforms and businesses from all sectors of the economy. Of them, our approach identified four of Nike’s biggest competitors, five Nike sub-brands and a major retailer of Nike products as the most associated accounts. This is consistent with our assertion that the Jaccard similarity of neighborhood sets provides a robust similarity measure between accounts. We found similar trends throughout the data. This observation is consistent with the experience of analysts at Starcount, a London-based social media analytics company, who are using the tool. Table 4 also shows how the size of the minhash signature affects the Jaccard estimate and the corresponding rank of similar accounts. We measure the Spearman rank correlation between the true Jaccard similarities (column *R*) and those calculated from signatures of length 100 (column ${\hat{R}}^{100}$) and 1000 (column ${\hat{R}}^{1000}$) to be 0.89 and 0.97 respectively. The close correspondence of the rank vector using signatures of length 1,000 and the true rank supports our decision to use signatures of containing 1,000 hashes.

**Table 4:** Twitter accounts with the highest Jaccard similarities to @Nike. *J* and *R* give the true Jaccard coefficient and Rank, respectively. $\hat{J}$ and $\hat{R}$ give approximations using Eq (3) where the superscript determines the number of hashes used. Signatures of length 1,000 largely recover the true Rank.

Twitter handle	*J*	*R*	${\hat{J}}^{100}$	${\hat{R}}^{100}$	${\hat{J}}^{1000}$	${\hat{R}}^{1000}$
adidas	0.261	1	0.22	2	0.265	1
nikestore	0.246	2	0.25	1	0.255	2
adidasoriginals	0.200	3	0.18	3	0.222	3
Jumpman23	0.172	4	0.13	7	0.166	4
nikesportswear	0.147	5	0.18	4	0.137	5
nikebasketball	0.144	6	0.16	5	0.127	7
PUMA	0.132	7	0.13	6	0.132	6
nikefootball	0.127	8	0.08	17	0.110	9
adidasfootball	0.112	9	0.09	16	0.113	8
footlocker	0.096	10	0.08	17	0.096	11

### Experiment 2: Comparison of community detection with PPR

In the following, we move from assessing a single component (minhashing) to system-wide experimentation: We evaluate the ability of our algorithm to detect related entities by measuring its performance as a local community detection algorithm seeded with members of ground-truth communities. As a baseline for comparison we use the PPR algorithm. PPR has been identified as the state of the art method for expanding communities from small seed sets by Kloumann and Kleinberg [27] who conducted a comprehensive assessment of local community detection algorithms on large graphs. In their study, Personal PageRank (PPR) [28] was the clear winner. In addition, they found that the improvement in performance of PPR asymptotes after three step random walks. Our PPR implementation uses the ground-truth seeds as the *teleport* set and runs for three iterations returning a ranked list of similar accounts.

To produce MS and AC results the seeds are input to an LSH query, which produces a set of candidate near-neighbors. For each candidate the Jaccard similarity is estimated using minhash signatures and the candidates are sorted by either the MS or AC procedures.

In all cases, we sequentially select accounts in similarity order and measure the recall after each selection. The recall is given by

$$\begin{array}{c} \text{recall}=\frac{\left|C\cap C_{\text{true}}\right|}{|C_{\text{true}}|-\left|S\right|} \end{array}$$ (17)

with *S* the initial seed set, *C*_true_ as the ground truth community and *C* as the set of accounts added to the output. For a community of size |*C*| we do this for the |*C*| − |*S*| most similar accounts so that a perfect system could achieve a recall of one.

#### Twitter dataset

In our experimentation with the Twitter dataset we randomly sample 30 seeds from each of the 16 ground-truth communities listed in Table 3. It is impossible to provide a fully like-for-like comparison with PPR: Running PPR on the full Twitter graph (700 million vertices and 20 billion edges) that we extract features from requires cluster computing and could return results outside of the hashed accounts. The alternative is to restrict PPR to run on the directly observed network of the 675,000 largest Twitter accounts, which could then be run on a single machine. We adopt this latter approach as it is the only option that meets our requirements (single machine and real-time).

We compare MS and AC to PPR operating on the directly observed network of the 675,000 largest accounts.

**Fig 12:**
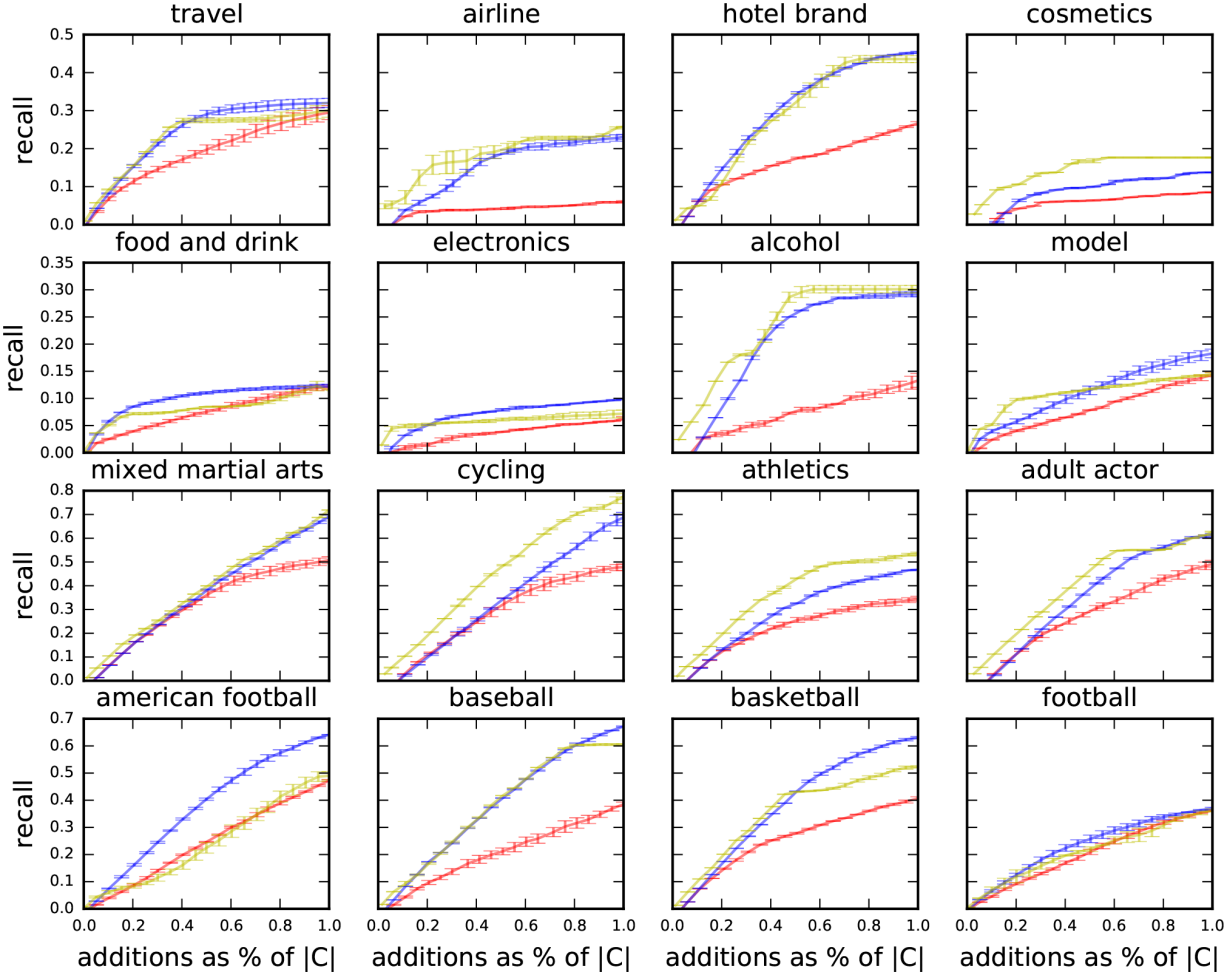
Twitter dataset average recall (with standard errors) of Agglomerative Clustering (yellow), Personal PageRank (red) and Minhash Similarity (blue) against the number of additions to the community expressed as a fraction of the size of the ground-truth communities given in Table 3. The tight error bars indicate that the methods are robust to the choice of seeds.

The results of this experiment are shown in Fig 12 with the Area Under the Curves (AUC) given in Table 5. Bold entries in Table 5 indicate the best performing method. In all cases, both MS and AC give superior results to PPR.

**Table 5:** Twitter dataset area under the recall curves (Fig 12). Bold entries indicate the best performing method. Minhash similarity (MS) is the best method in 8 cases, Agglomerative Clustering (AC) in 8 cases and Personalised PageRank (PPR) in none. A perfect community detector would score 0.5.

Tags	PPR	MS	AC
**travel**	0.186	**0.240**	0.230
**airline**	0.040	0.151	**0.180**
**hotel brand**	0.160	**0.294**	0.285
**cosmetics**	0.055	0.086	**0.143**
**food and drink**	0.072	**0.099**	0.082
**electronics**	0.035	**0.069**	0.059
**alcohol**	0.069	0.199	**0.229**
**model**	0.078	**0.110**	0.109
**mixed martial arts**	0.317	0.363	**0.386**
**cycling**	0.278	0.330	**0.445**
**athletics**	0.219	0.285	**0.365**
**adult actor**	0.269	0.347	**0.397**
**american football**	0.240	**0.371**	0.240
**baseball**	0.203	**0.379**	0.378
**basketball**	0.252	**0.380**	0.353
**football**	0.202	**0.233**	0.212

Fig 12 shows the average performance (including standard errors) over five randomly chosen input sets of 30 accounts from *C*_*true*_. The confidence bounds are tight indicating that the methods are robust to the choice of input seeds. Fig 12 is grouped according to the four types of ground-truth communities we discovered, where each type of ground-truth community exhibits different properties. Performance of all methods is considerable affected by the *quality* of the communities. Communities with good values of the metrics given in Table 3 in general have superior recall across all methods. The third row of Fig 12 contains the *best* communities as measured by the metrics in Table 3. For this group recalls are as high as 80% (Cycling, using agglomerative clustering). The worst group of communities are the transnational industrial communities in the second row. The lowest recall in row three (Athletics, using PPR) is still higher than the highest recall in the second row of results (Alcohol, using agglomerative clustering). The best performing method for every community in row three of the results is agglomerative clustering. The reason for this is that AC is an adaptive method that can incorporate information from early results. The downside of an adaptive method is that pollution from false positives can rapidly degrade performance. This can be seen in the steep decrease in gradient of the AC curves for basketball, baseball and adult actors. The fourth row of the table contains team sports. Team sports also have good metrics in Table 3, but differ markedly in structure from the communities in row three. The team sport communities have well-defined multi-modal sub-structures generated by the different teams. Both AC and MS are unimodal procedures that store the center of a set of data points. For a multi-modal distribution the mean may not be close to the distribution and so false positives will occur. As AC incorporates false positives into the estimation procedure for all future results MS outperforms AC for all team sport communities. Of the communities in the first and second rows of Fig 12 AC is best performing in 50% and MS is best performing in 50%. These communities are all diffuse, but some have a single densely connected region that can be found well by AC.

#### Email dataset

The email dataset contains 1005 vertices (email accounts), each of which is in exactly one of 42 communities (research departments of a large European research institute) and is available at http://snap.stanford.edu/data/email-Eu-core.html. Many of the communities are very small and so we only consider the largest 15 (those having more than 25 members). Due to the size of the dataset we use minhash signatures of length 100 instead of 1,000 and only seed the communities with 5 seeds.

**Fig 13:**
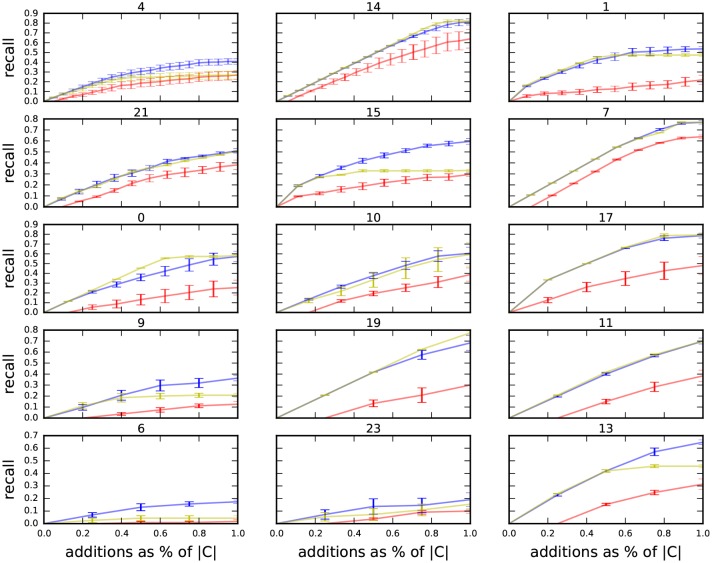
Email dataset average recall (with standard errors) of Agglomerative Clustering (yellow), Personal PageRank (red) and Minhash Similarity (blue) against the number of additions to the community expressed as a fraction of the size of the ground-truth communities given in Table 3. The tight error bars indicate that the methods are robust to the choice of seeds.

Fig 13 shows the average performance (including standard errors) over five randomly chosen input sets of five email accounts from *C*_*true*_. The confidence bounds are tight indicating that the methods are robust to the choice of input seeds. Table 6 gives the area under the curves of Fig 13. Again MS and AC outperform PPR in all cases, but this time MS is the best method for the majority of communities (10 cases), while AC is only best in five.

**Table 6:** Email dataset area under the recall curves (Fig 13). Bold entries indicate the best performing method. Minhash similarity (MS) is the best method in 10 cases, Agglomerative Clustering (AC) in 5 cases and Personalized PageRank (PPR) in none. A perfect community detector would score 0.5.

Tags	PPR	MS	AC
**4**	0.162	**0.271**	0.204
**14**	0.344	0.461	**0.473**
**1**	0.121	**0.382**	0.372
**21**	0.206	**0.306**	0.298
**15**	0.186	**0.396**	0.274
**7**	0.346	**0.450**	0.447
**0**	0.127	0.332	**0.384**
**10**	0.178	**0.345**	0.318
**17**	0.267	0.496	**0.503**
**9**	0.057	**0.210**	0.150
**19**	0.123	0.359	**0.383**
**11**	0.156	0.354	**0.361**
**6**	0.007	**0.102**	0.030
**23**	0.044	**0.103**	0.072
**13**	0.139	**0.357**	0.306

No mapping is provided from community labels to research departments and so for this dataset it is not possible to interpret the results in relation to the semantics of the community label.

#### Discussion

Much of the difference in performance of these methods derives from their respective ability to explore the graph: PPR is really a global algorithm that has been modified to find local relationships. After three iterations PPR uses first, second and third-order connections. First-order connection methods just use edges that directly connect to the seed nodes (neighbors). Second-order methods also give weight to the connections of the first-order nodes (neighbors of neighbors) and so on for third-order connections. The ability to explore higher-order connections is the principal reason identified by Kloumann [27] for the state-of-the-art performance of PPR. They also note that after two iterations most of the benefit is realized, and that after three iterations there is no more improvement.

Our implementations of MS and AC are effectively second-order methods since they operate on a derived graph where the edge weight between two vertices is calculated from the overlap of the respective neighborhoods. MS and AC outperform PPR because they are based on many more second-order connections than PPR: They run on a compressed version of the full graph instead of a sub-graph. PPR is expected to perform better given more computational resources, but the additional complexity, run time, latency or financial cost required for any scaled up/out solution will violate our system constraints.

#### Runtime analysis

Table 7 gives the mean and standard deviation of the algorithm run times averaged over the 16 communities. MS is the fastest method by two orders of magnitude. Average human reaction times are approximately a quarter of a second. Therefore, MS delivers a real-time user experience [1]. As MS is the only method capable of operating in the real-time domain. Since this is a system requirement, we choose the MS procedure for experiment 3 and in our operational prototype.

**Table 7:** Clustering runtimes averaged over communities.

Method	Mean(s)	Std.Dev.
PPR	12.58	8.83
MS	0.23	0.08
AC	18.6	22.0

### Experiment 3: Real-time graph analysis and visualization

**Fig 14:**
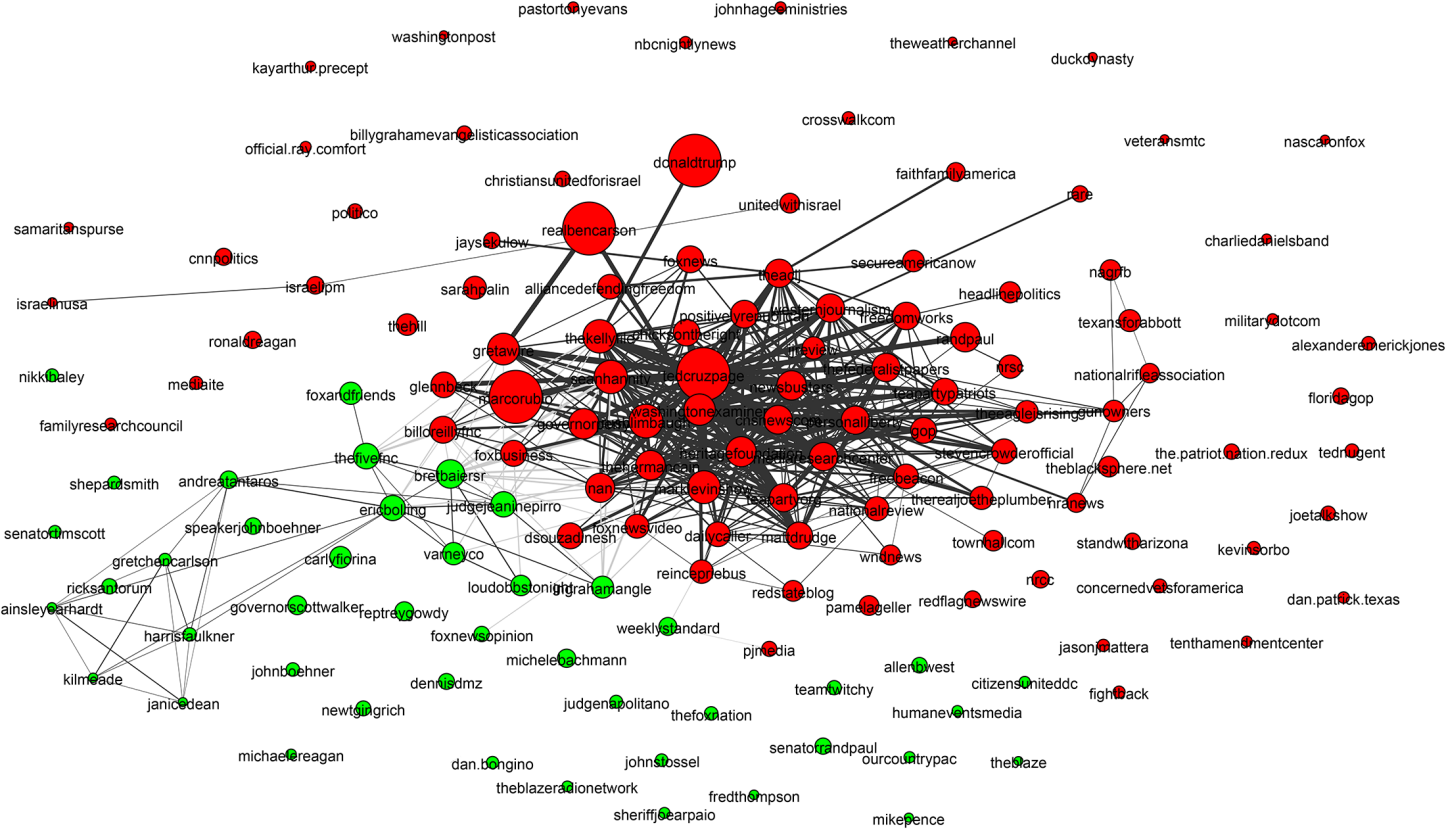
Communities around seeds from the US republican party in December 2015. Seeds are “Donald Trump”, “Marco Rubio”, “Ted Cruz”, “Ben Carson” and “Jeb Bush”. The vertex size depicts degree of similarity to the seeds. Edge widths show pairwise similarities. Colors are used to show different communities.

**Fig 15:**
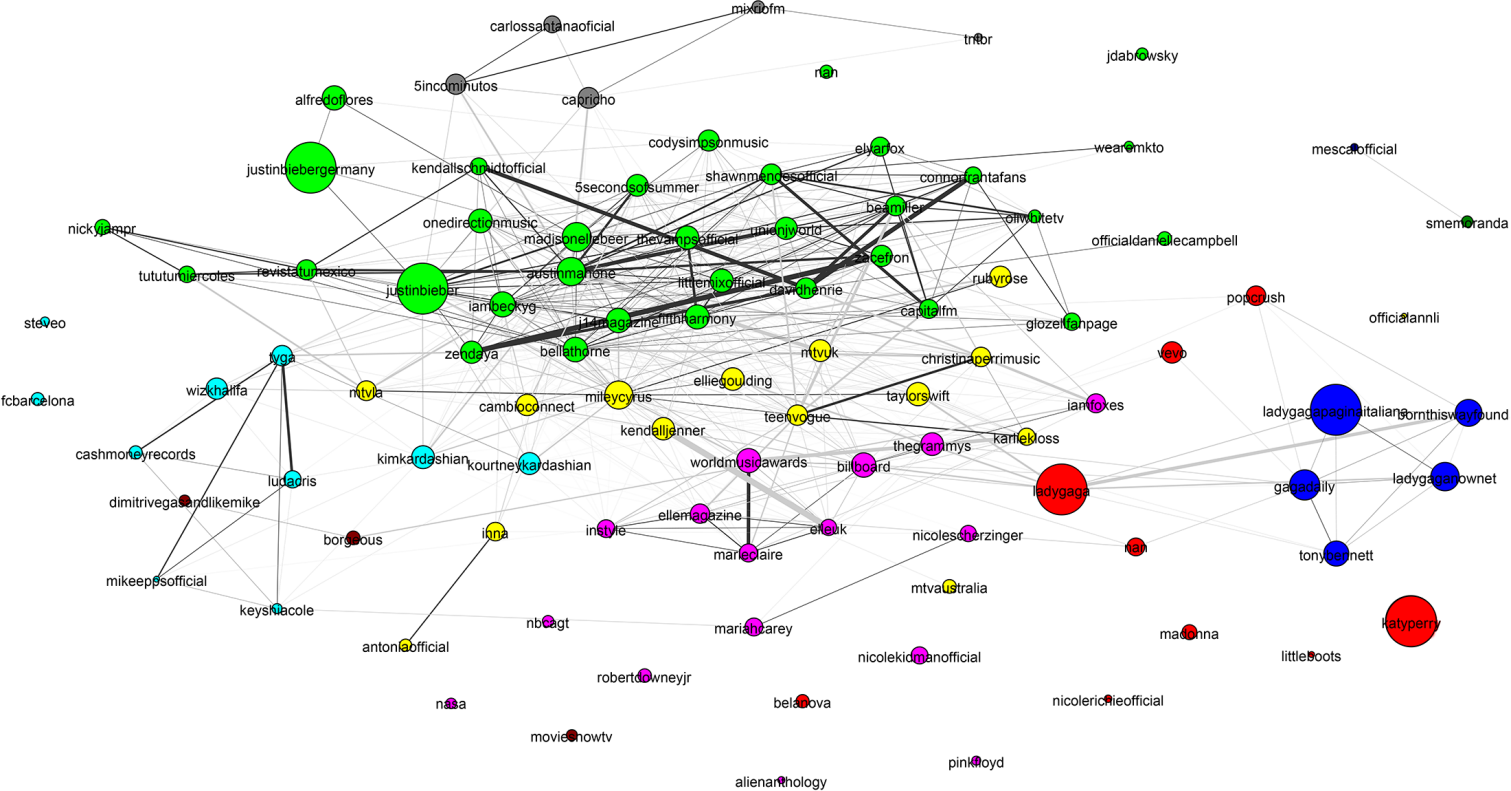
Visualization the Twitter Follower graph around global pop music. Seeds are “Justin Bieber”, “Lady Gaga” and “Katy Perry”. Vertex size depicts degree of similarity to the seeds. Edge widths show pairwise similarities. Colors represent different communities.

**Fig 16:**
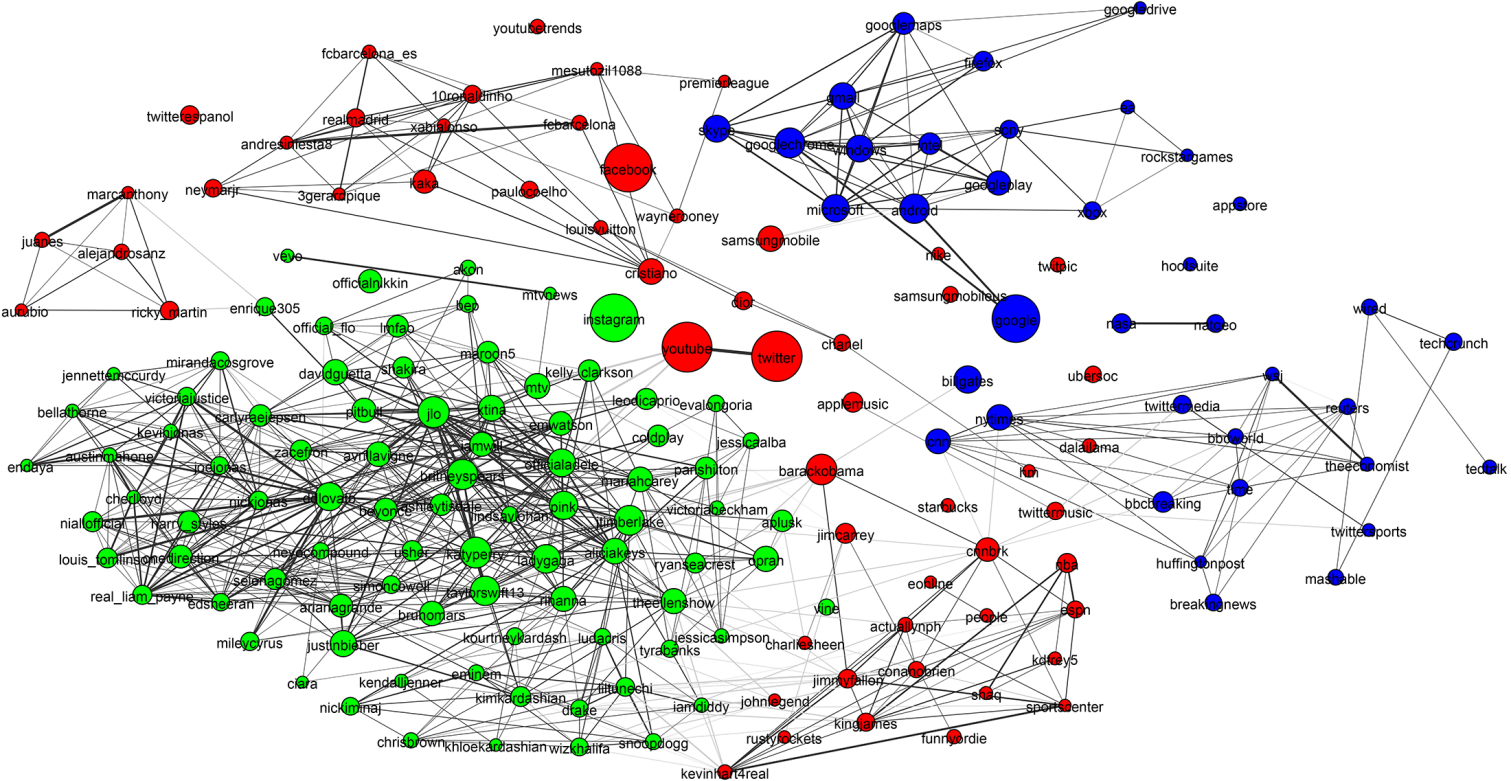
The major social networks. Seeds are Twitter, Facebook, YouTube and Instagram.

**Fig 17:**
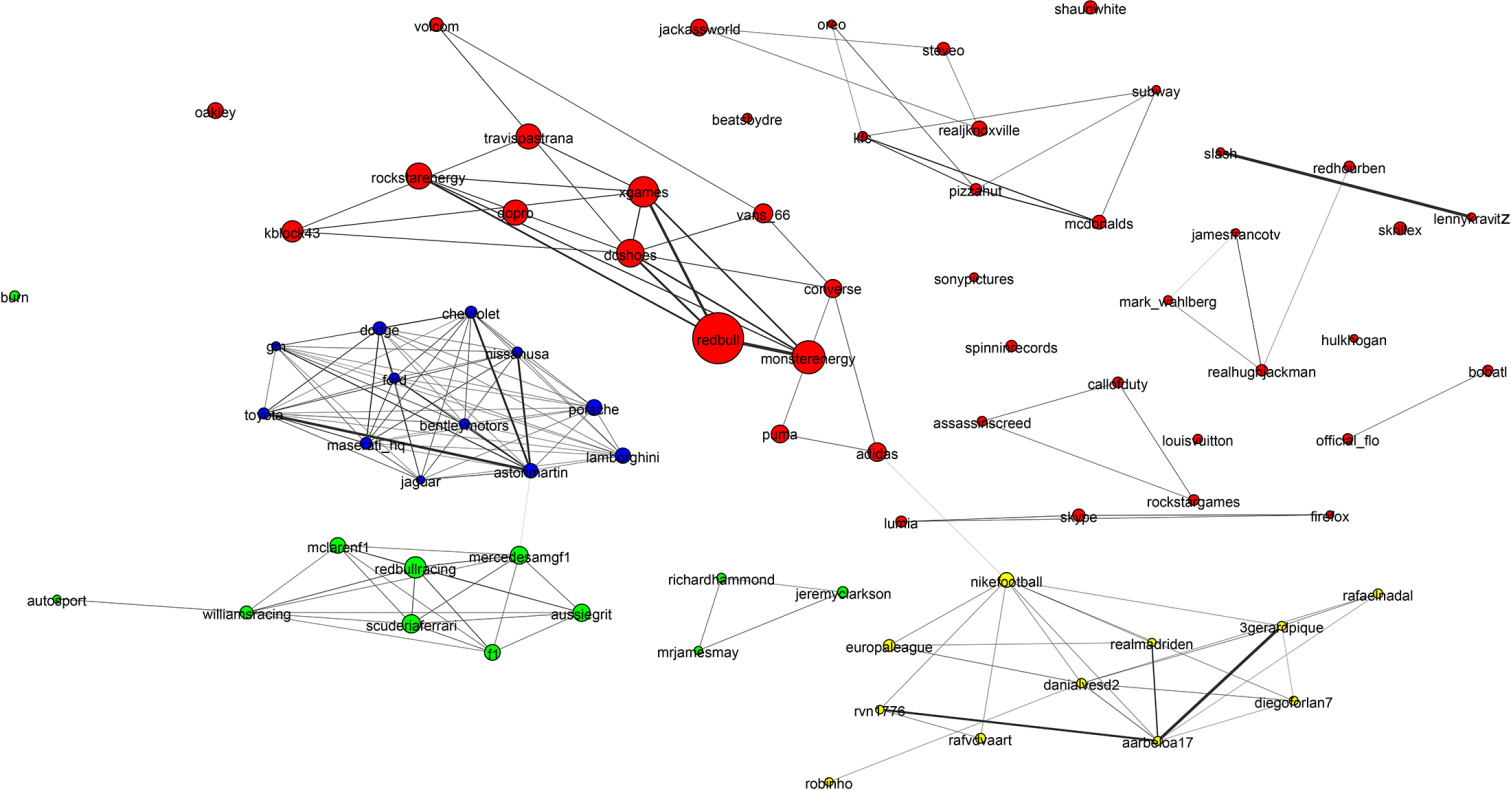
The many faces of RedBull.

**Fig 18:**
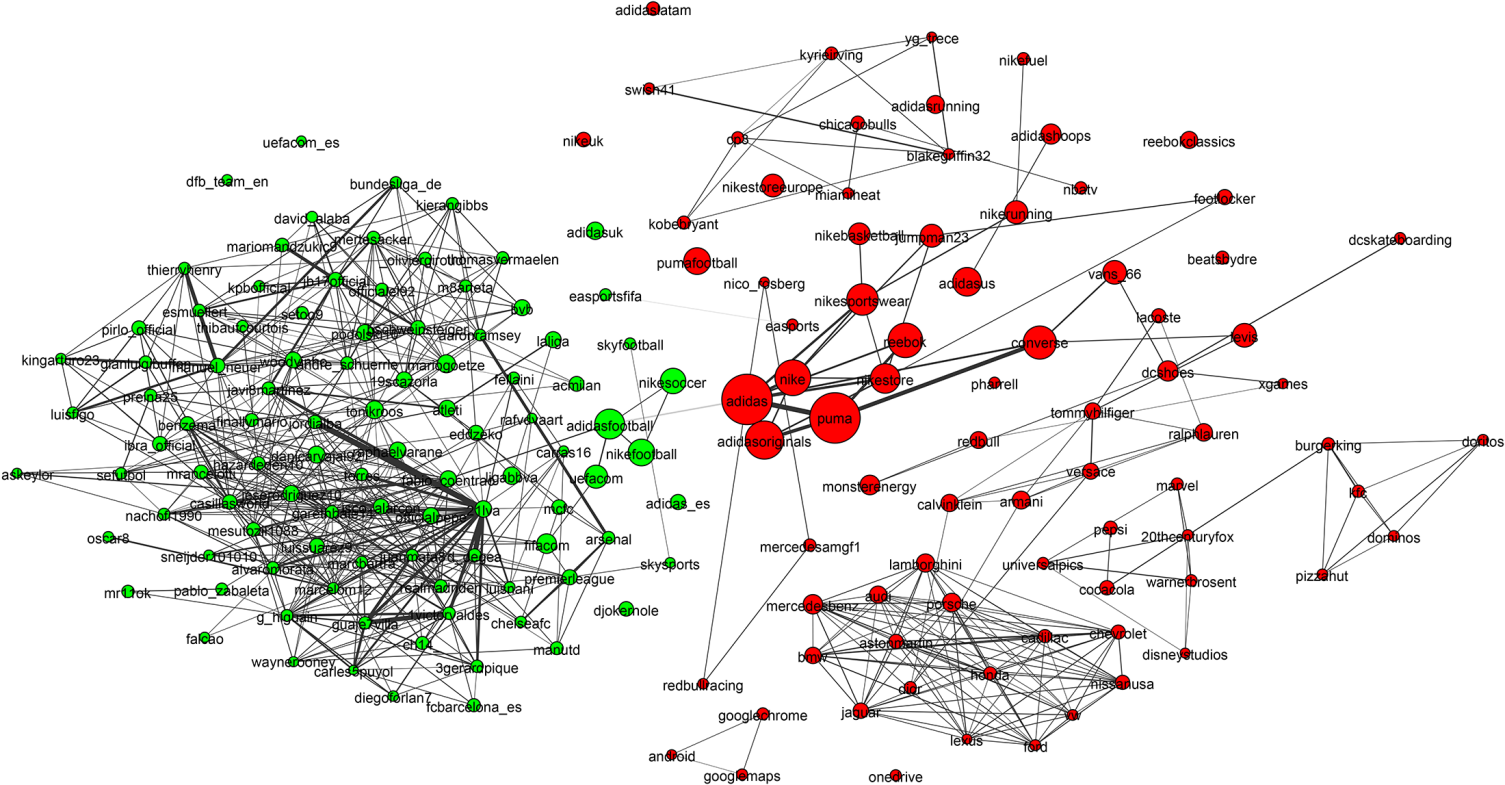
Visualisation of the Twitter graph around European sport brands. Vertex size depicts degree of similarity to the seeds. Edge widths show pairwise similarities. Colors represent different communities. Seeds are Adidas and Puma.

**Fig 19:**
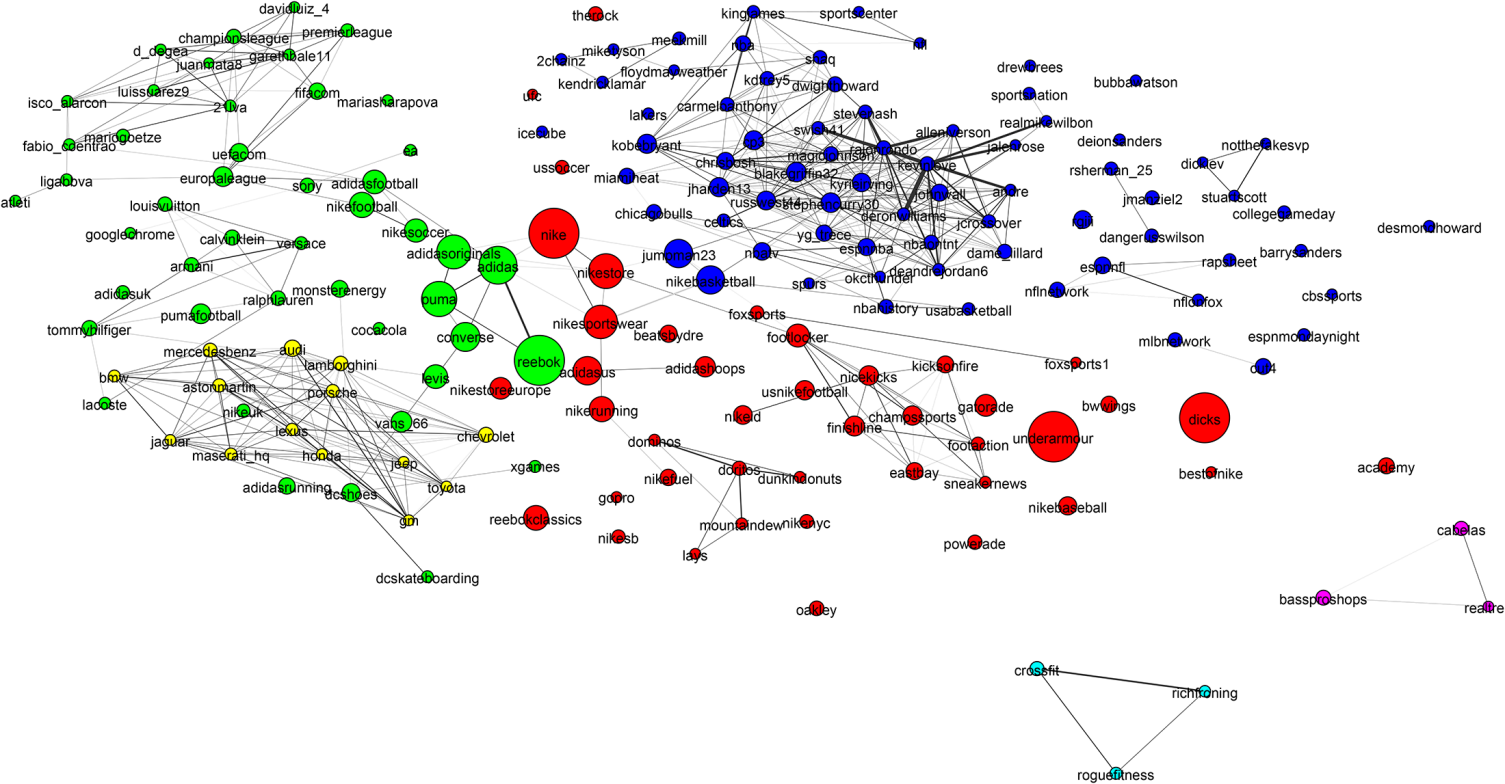
US sports brands. Seeds are Nike, Reebok, UnderArmour, Dicks.

In the following, we provide example applications of our real-time community detection system to graph analysis. Users need only provide a set of seeds, wait for a fraction of a second, and the system discovers the structure of the graph in the region of the seeds. Users can then interactively explore the discovered communities by providing different seeds based on what previous outputs reveal about the graph structure. Figs 14 and 15 show results on the Facebook Page Engagements network while Figs 16, 17, 18 and 19 use the Twitter Followers graph.

Each diagram is generated by the procedure shown in Fig 5: Seeds are passed to the MS process, which returns the 100 most related entities. All pairwise Jaccard estimates are then calculated using the minhash signatures. The resulting weighted adjacency matrix is passed to the WALKTRAP global community detection algorithm. The result is a weighted graph with community affiliations for each vertex. In our visualizations, we use the Force Atlas 2 algorithm to lay out the vertices. The thickness of the edges between vertices represents the pairwise Jaccard similarity, which has been thresholded for image clarity. The vertex size represents the weighted degree of the vertex, but is logarithmically scaled to be between 1 and 50 pixels. The vertex colors depict the different communities found by the WALKTRAP community detection algorithm.

We show some results using the Facebook Pages engagement graph to demonstrate that our work is broadly applicable across digital social networks. However, there are some key differences between the Facebook Pages engagement graph and the Twitter Followers graph. As Following is the method used to subscribe to a Twitter feed, Follows tend to represent genuine interest. In contrast, Facebook engagement is often used to grant approval or because a user desires an association. In addition, the Twitter graph corresponds to actions occurring as far back as 2006 (relatively few edges are ever deleted), while the Facebook graph corresponds only to events since 2014, when we began collecting data. As a result, the Twitter data set contains significantly more data, but with less relevance to current events.

Our work uses the vast scale and richness of social media data to provide insights into a broad range of questions. Here are some illustrative examples:

- **How would you describe the factions and relationships within the US Republican party?** This is a question with a major temporal component. Therefore, we use the Facebook Pages graph. We feed “Donald Trump”, “Marco Rubio”, “Ted Cruz”, “Ben Carson” and “Jeb Bush” as seeds into the system and wait for 0.25 s for Fig 14, which shows a densely connected core group of active politicians with Donald Trump at the periphery surrounded by a largely disconnected set of right-wing interest bodies. Note that our data set is from 12/2015, and Donald Trump’s community structure will have changed significantly by now.
- **Which factions exist in global pop music?** We feed the seeds “Justin Bieber”, “Lady Gaga” and “Katy Perry” into the system loaded with the Facebook Pages engagement graph and wait for 0.25 s for Fig 15, which shows that the industry forms communities that group genders and ethnicities.
- **How are the major social networks used?** We feed the seeds “Twitter”, “Facebook”, “YouTube” and “Instagram” into the system loaded with the Twitter Followers graph and wait for 0.25 s for Fig 16, which shows that Google is highly associated with other technology brands. Instagram is closely related to celebrity while YouTube and Facebook are linked to sports and politics.
- **How is the brand RedBull perceived by Twitter users?** We feed the single seed “RedBull” into the Twitter Followers graph and wait for 0.25 s for Fig 17, which shows that RedBull has strong associations with motor racing, sports drinks, extreme sports, gaming and football.
- **How does sports brand marketing differ between the USA and Europe?** We use the Twitter Followers graph. “Adidas” and “Puma” are the seeds for the European brands while “Nike”, “Reebok”, “UnderArmour” and “Dicks” are used to represent the US sports brands. Figs 18 and 19 show the enormous importance of football (soccer) to European sports brands, whereas US sports brands are associated with a broad range of sports including hunting, NFL, basketball, baseball and mixed martial arts (MMA).

In all cases, the user selects a group of seeds (or a single seed) and runs our system, which returns a figure and a table of community memberships in real-time (i.e., within 0.25 s). Analysts can then use the results to supplement the seed list with new entities or use the table of community members from a single WALKTRAP sub-community to explore higher resolution.

Similar tasks are traditionally conducted with techniques that are expensive and difficult to scale, e.g., telephone polling and focus groups, which often take months to return results. In contrast, our system provides an automatic analysis in the fraction of a second and at minimal cost, which allows for interactive community detection and exploration in large social networks.

## Conclusion and future work

We have presented a real-time system to automatically detect communities in large social networks. The system is computationally and memory efficient that it runs on a standard laptop. This work represents a technical advance leading to performance gains that are useful in practice and contains a rigorous evaluation on large social media data sets. The key contributions of this article are to demonstrate that (1) using the Jaccard similarity of neighborhood graphs provides a robust association metric between vertices of noisy and sparsely connected social networks; (2) Working with minhash signatures of the neighborhood graph dramatically reduces the space and time requirements of the system with acceptable approximation error; (3) Applying Locality Sensitive Hashing allows for approximate local community detection on very large graphs in real-time with an acceptable approximation error. For interactive and real-time community detection, we have demonstrated that our system finds higher quality communities in less time than the state-of-the-art algorithm operating under the constraints of a laptop. Our work has clear applications for interactive knowledge discovery processes that currently rely upon slow and expensive manual procedures, such as focus groups and telephone polling. In general, our system offers the potential for organizations to rapidly acquire knowledge of new territories and supplies an alternative monetization scheme for data owners.

In this article, we focused on digital social networks, but our method is applicable to all large networks including bipartite networks. The user-item bipartite networks that are studied in the field of recommender systems would be particularly amenable to this treatment, where items could be compactly modeled as minhash signatures of the users who have purchased them.

We leave two extensions for future work: First, currently, we treat the input social network as binary. In many settings, information is available to weight the edges. This might include message counts, the time since a connection was established or the type of connection. Efficient methods already exist for working with minhashes of weighted sets [40]. Therefore, an interesting progression of this work is to incorporate data with edges that can contain counts, weights and categorizations. The second extension incorporates some of the latest developments in the theory of minhashing. b-bit minhashing and Odd Sketches provide two promising approaches to extend our system to even larger graphs [42, 43]. Both offer the best cost/benefit trade-off when sets are very similar (Jaccard similarity ≈ 1) or when sets contain most of the elements in the sample space. DSN data typically contains sets that are very small relative to the sample space and with Jaccard similarities ≪0.5. As an example, our Twitter data has a sample space containing 7 × 10^8^ elements with a typical set containing 10^4^ elements. The strong theoretical bounds of these algorithms do not hold in these DSN-typical settings. Therefore, a cost/benefit analysis would be required before implementing either in an extension.

## Supporting information

S1 File Supporting material describing the the data acquisition methodology and given detailed axiomatic definitions of good communities. (PDF)
