# Supplementary material for: Real-time community detection in full social networks on a laptop
Source: PLoS One. 2018 Jan 17;13(1):e0188702. doi: 10.1371/journal.pone.0188702 (PMC5771570; doi:10.1371/journal.pone.0188702)
Supplement: S1 File — (PDF) [file pone.0188702.s001.pdf]

Supporting Information

Efficient Minhash Generation

A naive Python implementation for generating minhash signatures requires six days to run on a desktop computer with 6 physical (12 logical) Intel i7 5930k @3.5GHz cores and 64GB of RAM. This is prohibitive for nightly updates and so we highly optimized this part of the code base. The code was ported to the Python-to-C bridge project, Cython, which allowed us to add type information and compiler directives to turn off array bounds checking (there is a large amount of array dereferencing). We stored the input matrices in contiguous memory, removing any superfluous code (logging, most inline error checking). The loops were then rewritten to be vectorized by the SIMD processor. The fully optimized Cython version of the minhash implementation runs (in parallel on 6 cores) in approximately one hour.

Crawling Social Networks

Most providers of social networks offer public Application Programming Interfaces (APIs) to their networks. By doing so they allow 3rd party developers to build applications using their data. This stimulates innovation and embeds their network more deeply into the everyday lives of its users, and in cases of large networks, global social and economic life. Delivering data at massive scale can incur significant costs. To manage these, all networks limit the rate at which data can be downloaded through their APIs.

Rate limiting varies between networks, but most work on a per user basis. Doing this ensures that larger more popular apps are able to get the data they need. A user access token is granted whenever a user agrees to sign up to an app using Facebook / Twitter etc. This work makes use of several client facing applications to provide user access tokens (Starcount Playlist, Starcount Vibe and Chatsnacks).

To optimize data throughput while remaining within the DSN rate limits we developed an asynchronous distributed network crawler using Python's Twisted

library [1]. The crawler consists of a server responsible for token and work management and a distributed set of clients making http requests to DSNs (see Fig 1).

Fig 1. the distributed asynchronous system built for data acquisition from digital social networks.

The server contains a credential manager that holds access tokens in a queue and monitors the number of calls to each API. Once a token has been exhausted it is put to the back of the queue and locked until its refresh time. The server communicates over TCP with the clients responding to requests for work and access tokens with account ids and fresh access tokens/pause responses respectively:

The clients make asynchronous requests to the DSNs, handling response codes, parsing and storing data. A conventional program will *block* while waiting for an http response. When the principal function of a program is to download data, blocking time amounts to the vast majority of the run time. One solution is to run the program using multiple threads. However, for this application threads carry an unnecessary overhead and induce inefficiencies as data is naively moved between caches by the operating system. The asynchronous programming paradigm offers a superior alternative to explicit multi-threading. Asynchronous programming makes use of an event loop that constantly *listens* for new jobs and does not block while waiting for http responses.

We originally implemented the system using an 80 MB shared fiber optic connection, but our downloads caused network blackouts. Therefore, we designed a distributed system that could be partially deployed in the cloud. The final system is depicted in Fig 1. The access tokens and account IDs to query (work) live on a server on our local network. Clients are deployed to Amazon's elastic cloud from where all interactions with DSN servers occurs. We configured the clients to establish persistent connections to the API endpoints. Every time a connection is opened, a handshake must occur. For secure systems (communicating over https), the handshake is particularly onerous, requiring the exchange of security certificates.

Community Axioms

Homophily only applies to attributes that ease information flow between individuals. Some attributes have no effect or are divisive (for instance right-handed people feel no

sense of kinship) and so should not be associated with communities. Additionally attributes may be at the wrong scale to describe structural sub-units (sports person rather than footballer). A community evaluation based on ground-truth that were not communities would have no value. We apply community goodness functions to each prospective 'tag community' to identify to what extent these functional traits generate structurally observable communities.

For each functional group we generate the complete weighted intersection graph by calculating all pairwise Jaccard similarities and evaluate the six metrics in Table 3. They are adapted from [2] to apply to weighted graphs. As we work with a derived graph where each edge weight is the Jaccard similarity of neighborhoods, the metrics have slightly different interpretations. Two entities in the derived graph are strongly connected if they have very similar neighborhoods. Since for the large entities the neighborhood normally has at least an order of magnitude more incoming than outgoing edges, entities are closely related if they have a similar fan/follower base. We define S to be the set of vertices comprising a community and a weighted graph $G(V, E, W)$ where W is a weight matrix. The internal edge weight of S is

$$m_s = \sum_{\{i,j \in S\}} W_{i,j} \,, \tag{1}$$

and the weight of edges that cross the boundary of S is

$$c_s = \sum_{\{i \in S, j \notin S\}} W_{i,j} \,. \tag{2}$$

The community goodness metrics are then given by:

- **Clustering** exploits the idea that people in communities are likely to introduce their friends to each other. It measures how cliquey a community is. In our paradigm clustering is high if followers of a community recommend things for other followers of the community to like or follow. If a vertex has k_n neighbors then $\frac{1}{2}k_n(k_n - 1)$ possible connections can exist between the neighbors. The clustering of a node gives the fraction of its neighbors' possible connections that exist. The clustering of a community is the average clustering of each vertex. Clustering is sometimes referred to as the proportion of triadic closures in the

network. The weighted clustering of the ith vertex is given by

$$Cl_i(S) = \frac{W_s^3}{(W_s W_{\max} W_s)_{ii}} \tag{3}$$

where $W_{\max}$ is a matrix where each entry is the maximum weight found within S [3].

- **Conductance** is an electrical analogy for how easily information entering the community can leave it. In our context, it is defined as

$$Con(S) = \frac{c_s}{2m_s + c_s}\,, \tag{4}$$

i.e., it is the ratio of the community's external to total edge weight. A low value means that the the community is well separated from the rest of the network. In our paradigm, conductance is low if the followers of the community are not interested in other communities.

- **Cohesiveness** measures how easily the community can be split into disconnected components. A good community is not easily broken up. The cohesiveness is given by the minimum conductance of any sub-community. A low value indicates a bad community as there is at least one well-separated sub-community. In our paradigm, low cohesiveness corresponds to members of the community having distinct, non-overlapping follower groups.

$$Coh(S) = \min_{\{S' \subset S\}} Con(S') \tag{5}$$

Iterating through all subsets S' of S is impractical. Thus, we sample S' by randomly selecting 10 subsets of starting vertices, running PPR community detection for each and taking a sweep through the PageRank vector to find the minimum conductance cut.

- **Conductance Ratio (CR)** is the ratio of conductance to cohesiveness and

defined as

$$CR(S) = \frac{Con(S)}{Coh(S)}. \quad (6)$$

A large number indicates that the community could be broken up into structural sub-units.

- **Density** is given by the ratio of the community's total internal edge weight to the maximum possible if every edge was present with weight one:

$$Den = \frac{2m_s}{n_s(n_s - 1)} \quad (7)$$

A high number indicates a highly interconnected community. In our paradigm, this corresponds to a community with a well-defined follower base that is interested in most community members.

- **Separability** measures how well the community is separated from the rest of the network. It is the ratio of internal to external edges and so is closely related to conductance:

$$Sep(S) = \frac{m_s}{c_s} \quad (8)$$

In our paradigm, a high value indicates that followers of the community are not interested in much else.

References

1. Wysocki R, Zabierowski W. Twisted framework on game server example. 2011 11th International Conference The Experience of Designing and Application of CAD Systems in Microelectronics. 2011; p. 361–363.

2. Yang J, Leskovec J. Overlapping community detection at scale: a nonnegative matrix factorization approach. In: Proceedings of the 6th international conference on Web search and data mining. ACM; 2013. p. 587–596.

3. Holme P, Min Park S, Kim BJ, Edling CR. Korean university life in a network perspective: Dynamics of a large affiliation network. Physica A: Statistical mechanics and its Applications. 2007;373:821–830.
